# Polyamine metabolism links gut microbiota and testicular dysfunction

**DOI:** 10.1186/s40168-021-01157-z

**Published:** 2021-11-11

**Authors:** Qi Zhao, Jian-Feng Huang, Yan Cheng, Man-Yun Dai, Wei-Feng Zhu, Xiu-Wei Yang, Frank J. Gonzalez, Fei Li

**Affiliations:** 1 Frontiers Science Center for Disease-related Molecular Network, State Key Laboratory of Biotherapy, West China Hospital, Sichuan University, Chengdu, 610041 China; 2 State Key Laboratory of Phytochemistry and Plant Resources in West China, Kunming Institute of Botany, Chinese Academy of Sciences, Kunming, 650201 China; 3 Shanwei Institute for Food and Drug Control, Shanwei, Guangdong Province 516622 China; 4 Academician Workstation, Jiangxi University of Traditional Chinese Medicine, Nanchang, 330004 China; 5 School of Pharmaceutical Sciences, Peking University Health Science Center, Peking University, Beijing, 100191 China; 6 Laboratory of Metabolism, Center for Cancer Research, National Cancer Institute, National Institutes of Health, Bethesda, MD 20892 USA

**Keywords:** Testicular dysfunction, Gut microbiota, Polyamine metabolism, Metabolomics

## Abstract

**Background:**

Male fertility impaired by exogenous toxins is a serious worldwide issue threatening the health of the new-born and causing infertility. However, the metabolic connection between toxic exposures and testicular dysfunction remains unclear.

**Results:**

In the present study, the metabolic disorder of testicular dysfunction was investigated using triptolide-induced testicular injury in mice. We found that triptolide induced spermine deficiency resulting from disruption of polyamine biosynthesis and uptake in testis, and perturbation of the gut microbiota. Supplementation with exogenous spermine reversed triptolide-induced testicular dysfunction through increasing the expression of genes related to early and late spermatogenic events, as well as increasing the reduced number of offspring. Loss of gut microbiota by antibiotic treatment resulted in depletion of spermine levels in the intestine and potentiation of testicular injury. Testicular dysfunction in triptolide-treated mice was reversed by gut microbial transplantation from untreated mice and supplementation with polyamine-producing *Parabacteroides distasonis*. The protective effect of spermine during testicular injury was largely dependent on upregulation of heat shock protein 70s (HSP70s) both in vivo and in vitro.

**Conclusions:**

The present study linked alterations in the gut microbiota to testicular dysfunction through disruption of polyamine metabolism. The diversity and dynamics of the gut microbiota may be considered as a therapeutic option to prevent male infertility.

Video Abstract

**Supplementary Information:**

The online version contains supplementary material available at 10.1186/s40168-021-01157-z.

## Background

Infertility is a worldwide clinical issue affecting approximately 12% of the reproductive-aged couples, among which males contribute to nearly 50% of all cases [1]. Moreover, the risks of deformities and defects for the new-born caused by dysfunctional male fertility are also critically important. Growing evidence revealed that in recent decades the sperm quality of men has declined in both industrialized and developing countries, which has raised concerns from the academic community to whole society [1, 2]. Accordingly, most cases of abnormal male reproduction in humans, except for genetic defects, are derived from exogenous chemical exposures, including endocrine disrupters and therapeutic drugs, particularly for chemotherapy [3, 4]. It was firmly established that chemical exposures impair male fertility by damaging testicular cells and the hormonal environment, resulting in decreased semen quality and testicular dysfunction [5].

Triptolide (TP) is a diterpene that was extracted from *Tripterygium wilfordii* Hook F in 1972, and is widely used as antineoplastic, antispermatogenic, and immunosuppressive therapies. Testicular dysfunction is a common side-effect of TP and its commercial drugs both in rodent experimental models and humans [6]. Continuous administration of TP significantly suppresses the marker-enzymes of spermatogenesis and testosterone levels, reduces sperm counts, diminishes the testis indices (testis weight/body weight × 100%), and damages the microstructure of testis in mice [7]. The severe testicular toxicity induced by TP largely limited its clinical use in humans despite its notable therapeutic effects on inflammatory, autoimmune diseases, and cancers [8, 9]. The mechanism underlying testicular toxicity triggered by this typical toxicity remains unclear. Oxidative stress and its activation of signaling pathways, such as the nuclear factor-E2-related factor 2 (Nrf2)-mediated antioxidant response were thought to be the major reason, which could be prevented by N-acetyl-L-cysteine and resveratrol treatment in mice [10]. Uncovering the testicular toxicity of TP was helpful to explore the process of male infertility.

Gut microbiota, the second largest genome of the host, was reported to impact the physiological function of liver, gut, brain, immune cells, and certain endocrine gland [11–17]. Gut microbiota plays an important role in testis. Disruption of the gut microbiota by di-(2-ethylhexyl) phthalate was shown to alter the male reproductive system in rats [18], and the gut microbiota also modulated the permeability of the blood-testis barrier and performed a role in the regulation of endocrine functions of the testis in mice [19]. Additionally, the gut microbiota may have potential role in the treatment of male infertility in a metabolic syndrome sheep model [20]. Spermine can be synthetized through endogenous polyamine metabolism and obtained from the dietary uptake and gut microbiota, such as *Actinobacteria*, *Firmicutes*, *Proteobacteria*, and *Bacteroidetes* [21]. The functions of spermine includes antioxidation, regulation of ion channels, inhibition of lipid synthesis, and maintaining the normal physiology of reproduction [22].

In the current study, spermine deficiency in testis and gut microbiota was closely linked to testicular dysfunction. Gut microbiota was found to play an important role in protecting against TP-induced testicular injury mainly through modulation of polyamine metabolism. Thus, caution for the clinical use of TP and its preparations is warranted. Furthermore, supplementation with spermine and the intervention of gut microbiota using prebiotics and probiotics may be a promising strategy to improve the function of testis.

## Results

### Testicular dysfunction accompanied by metabolic disorder

**Fig. 1:**
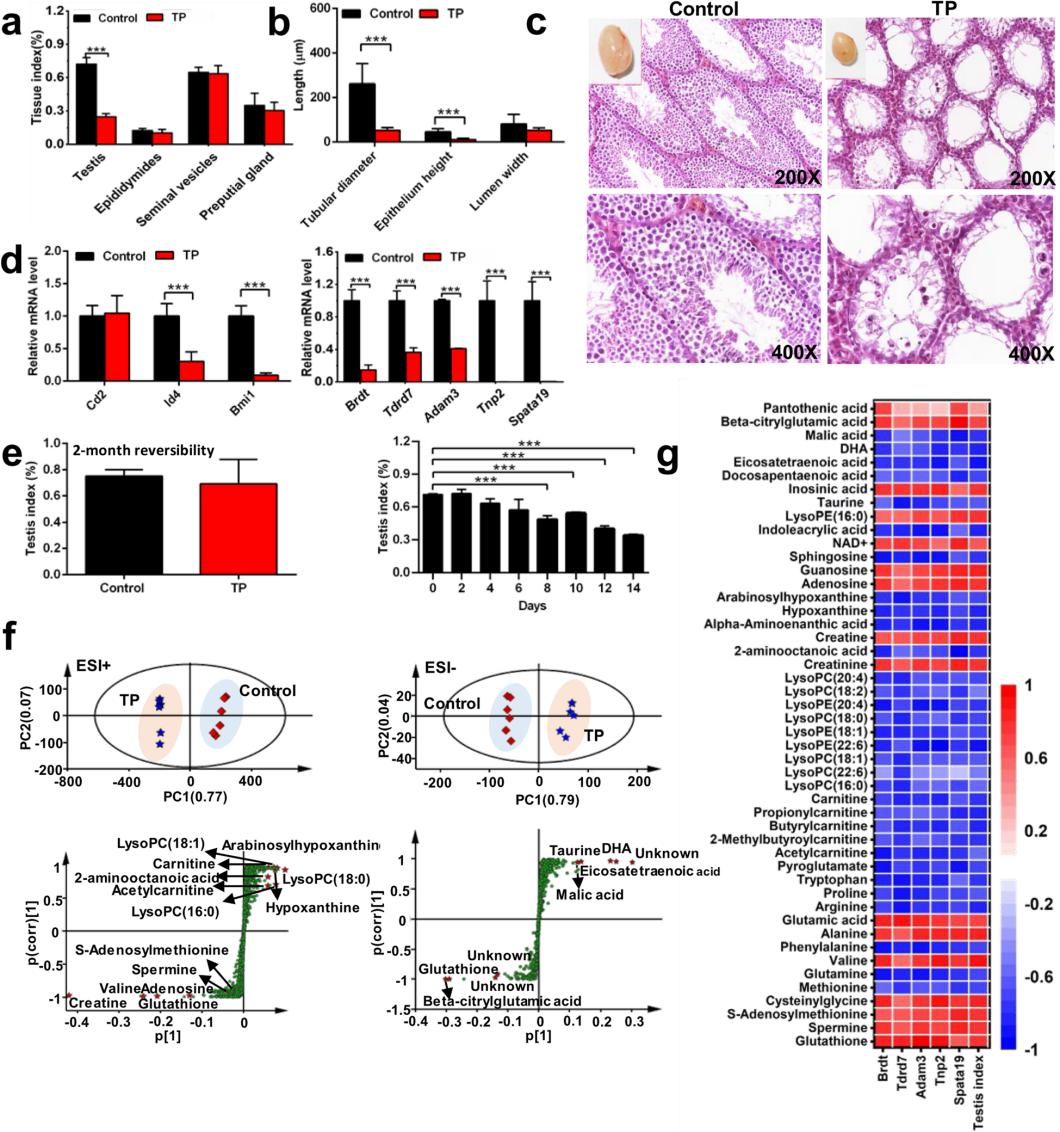
TP induced testicular toxicity and metabolic disruption. **a** Tissue index (tissue weight/body weight × 100%). **b**, **c** H&E staining, tubular diameter, epithelium height and lumen width of the testis. **d** mRNA level of genes involved in early and late spermatogenic events. **e** Reversibility and time-dependent effect of TP-induced testicular toxicity. **f** PCA score plot and OPLS-DA *S*-plot for testis metabolome detected in ESI+ and ESI− modes. **g** Spearman’s correlation between the altered metabolites and indicators of testicular injury. ***P < 0.001 (*n* = 6). NAD+: nicotinamide adenine dinucleotide; DHA: docosahexaenoic acid

TP caused severe testicular injury in mice (8- to 10 weeks old, 25–30 g) after intraperitoneal injection at 0.2 mg/kg for 14 days. Although the body weight and organ indices (organ weight/body weight × 100%) of epididymides, seminal vesicles, and preputial gland were maintained, the testicular index was markedly reduced after TP treatment (Fig. 1a and Supplementary Figure 1a). H&E staining and immunohistochemistry (IHC) showed that TP reduced the tubular diameter and epithelium height of testis and time-dependently attenuated the differentiated spermatogonium (marked by C-kit), meiotic spermatocyte (marked by synaptonemal complex protein 3 (Scp3)), late meiotic spermatocytes (marked by cyclic AMP-responsive element modulator (Crem)), round spermatid (marked by Crem and Acr), elongating spermatid (marked by Acr), and sertoli cell (marked by Vim) (Fig. 1b, c, e and Supplementary Figure 2). IHC showed that spermatogonial stem cell (marked by inhibitor of differentiation 4 (Id4)) decreased mildly, although the marks of spermatogonical stem cell *Id4* mRNA and B lymphoma Mo-MLV insertion region 1 (*Bmi1*) mRNA levels were decreased (Fig. 1d and Supplementary Fig. 2). Differentiated spermatogonium (marked by C-kit) was the most sensitive cell in our study, which was the first to disappear at day 6 after TP treatment (Supplementary Figure 2). Furthermore, TP reduced the expression of mRNAs involved in late spermatogenic events including bromodomain testis-specific factor (*Brdt*, involved in the generation of male gametes in post-meiotic cells), tudor domain-containing 7 (*Tdrd7*, involved in dynamic ribonucleoprotein remodeling of chromatoid bodies during spermatogenesis), a disintegrin and metallopeptidase domain 3 (*Adam3*, involved in sperm assembly and sperm-zona pellucida binding), transition protein 2 (*Tnp2*, involved in histone displacement), and spermatogenesis associated 19 (*Spata19*, involved in mitochondria adhesion of the sheath during spermatogenesis) mRNAs (Fig. 1d).

UPLC-QTOF-MS-based metabolomics was conducted to uncover the metabolic features of the mouse testis following testicular injury. Principal component analysis (PCA) and *S*-plot in both ESI+ and ESI− modes demonstrated that the TP group clearly separated from the control group as a result of several reduced metabolites, including glutathione, creatine, adenosine, S-adenosylmethionine, and spermine (Fig. 1f). VIP values and statistical significance of the altered metabolites were displayed in Supplementary Figure 1b. Pathway enrichment showed that glutathione metabolism, malate-aspartate shuttle, urea cycle, and spermidine and spermine biosynthesis were disrupted by TP (Supplementary Figure 1c). Detailed information for metabolite identification is shown in Supplementary Table 1. Spearman’s correlation analysis revealed a strong correlation between testicular injury and metabolites, including glutathione, adenosine, carnitines, valine, and compounds involved in spermine metabolism (Fig. 1g). These results showed that TP caused severe testicular injury and affected various metabolic pathways, especially for spermine metabolism.

### Polyamine metabolism was suppressed in both testis and gut microbiota following testicular injury

**Fig. 2:**
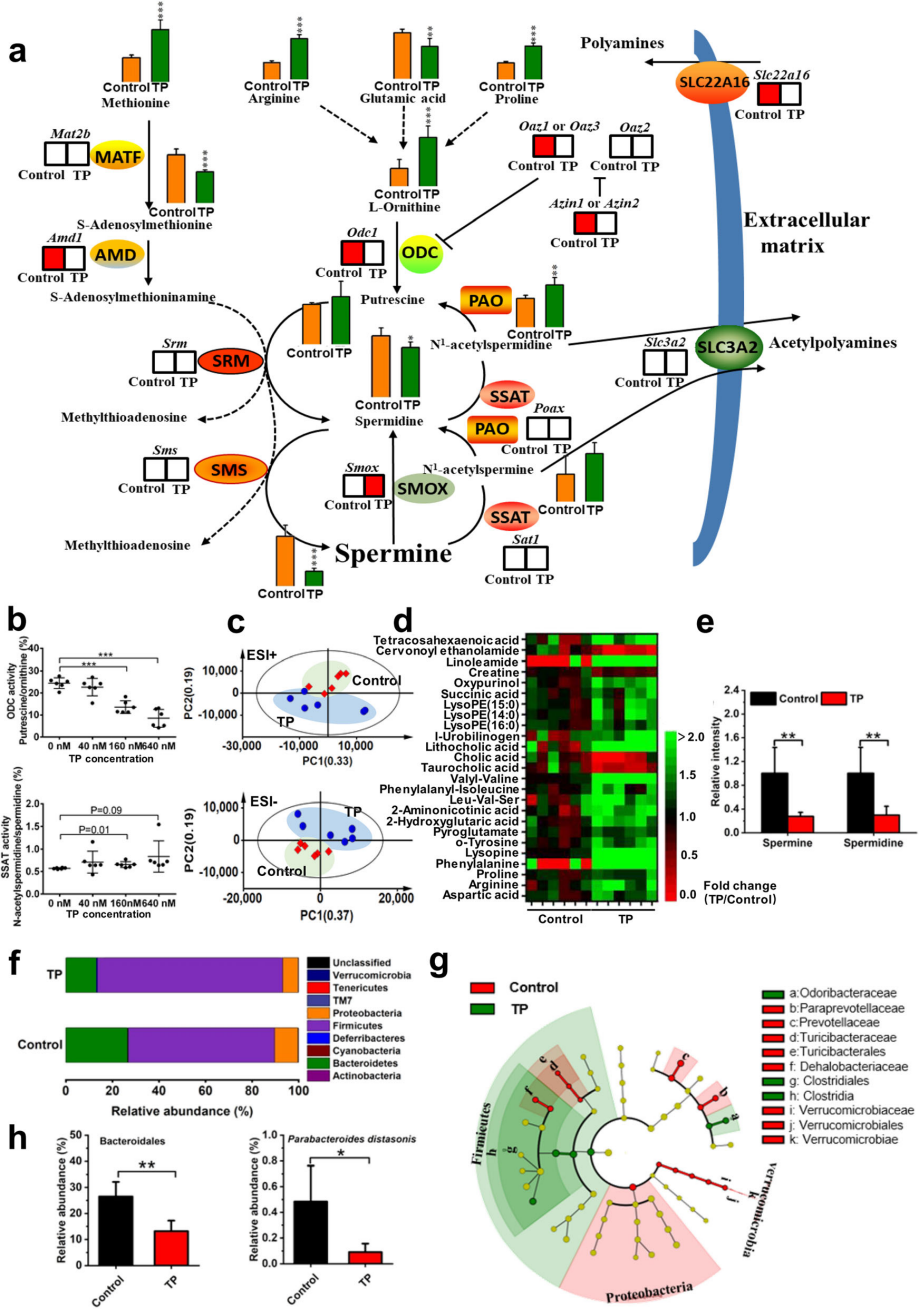
Polyamine metabolism was suppressed in both testis and gut microbiota following testicular dysfunction. **a** Pathway of spermine metabolism in testis (*n* = 6). **b** ODC and SSAT activities (*n* = 6). TP (0, 40, 160, 640 nm TP) was incubated with testicular extracts in vitro. **c** PCA score plot for cecum content metabolome (ESI+ and ESI− modes, *n* = 6). **d** Changed metabolites in cecum content include bile acid and amino acid (*n* = 6). **e** Spermine and spermidine levels in cecum content revealed by targeted analysis (*n* = 6). **f** Relative abundance of phylum in cecum content (*n* = 6). **g** Cladogram for gut microbiota (control group vs TP group, *n* = 6). **h** Relative abundance of advantaged germs in polyamine biosynthesis (*n* = 6). *P < 0.05, **P < 0.01, and ***P < 0.001

According to the metabolomics outcome of testis, further studies focused on spermine metabolism. Ornithine decarboxylase 1 (*Odc1*) and adenosylmethionine decarboxylase 1 (*Amd1*) participate in polyamine biosynthesis, which were inhibited by TP (Fig. 2a and Supplementary Figure 3a). *Odc1* regulating gene ornithine decarboxylase antizyme 1/3 (*Oaz1/3*) and antizyme inhibitor 1/2 (*Azin1/2*), as well as polyamine influx transporter (*Slc22a16*) mRNAs were also decreased following testicular injury (Fig. 2a and Supplementary Figure 3a). Conversely, the mRNA levels of spermine oxidase (*Smox*) gene encoding enzymes that convert spermine to spermidine were increased (Fig. 2a and Supplementary Figure 3a). Enzyme activity of ODC and spermidine/spermine-N^1^-acetyltransferase (SSAT) was measured to confirm their functions. ODC activity was inhibited significantly by TP, and SSAT activity could be increased slightly (Fig. 2b). The decrease in gene expression was also in concordance with the metabolome. The levels of substrates for polyamine synthesis including arginine, proline, and ornithine were increased in testis while spermine and spermidine were reduced after TP exposure (Fig. 2a and Supplementary Figure 3d). The MS/MS of spermine and spermidine are displayed in Supplementary Figure 3b. Correlation analysis found that spermine and spermidine levels were positively related to testis index (Supplementary Figure 3c).

Considering that gut microbiota-derived polyamines are a critical source for the host polyamine pool [23] and influence the physiology and disease of the host [24], the composition of gut microbiota and their metabolites were investigated. PCA score plots of cecum content showed that metabolite levels were markedly disrupted by TP treatment, including amino acids and bile acids (Fig. 2c, d), while spermine and spermidine were reduced after TP treatment (Fig. 2e). The identification of the altered metabolites is listed in Supplementary Table S2. Metagenomics revealed that TP raised the relative abundance of *Firmicutes* and reduced *Bacteroidetes* and *Proteobacteria* in cecum lumen (Fig. 2f, g). Except for phylum, microbial community in class, order, family, genus, and species were all disrupted by TP (Supplementary Figure 4a). Notably, *Bacteroidales* (order), *Parabacteroides* (genus), and *Parabacteroides distasonis* (species) belonging to *Bacteroidetes* were decreased following TP-induced testicular injury (Fig. 2h and Supplementary Figure 4b). *Parabacteroides distasonis* and most other strains of *Bacteroidetes* are involved in polyamine production [25]. Taken together, these data indicated that impaired polyamine biosynthesis of both testis and gut microbiota as well as the damaged polyamine uptake system in testis resulted from testicular dysfunction.

### Supplementation with polyamines ameliorated testicular dysfunction

**Fig. 3:**
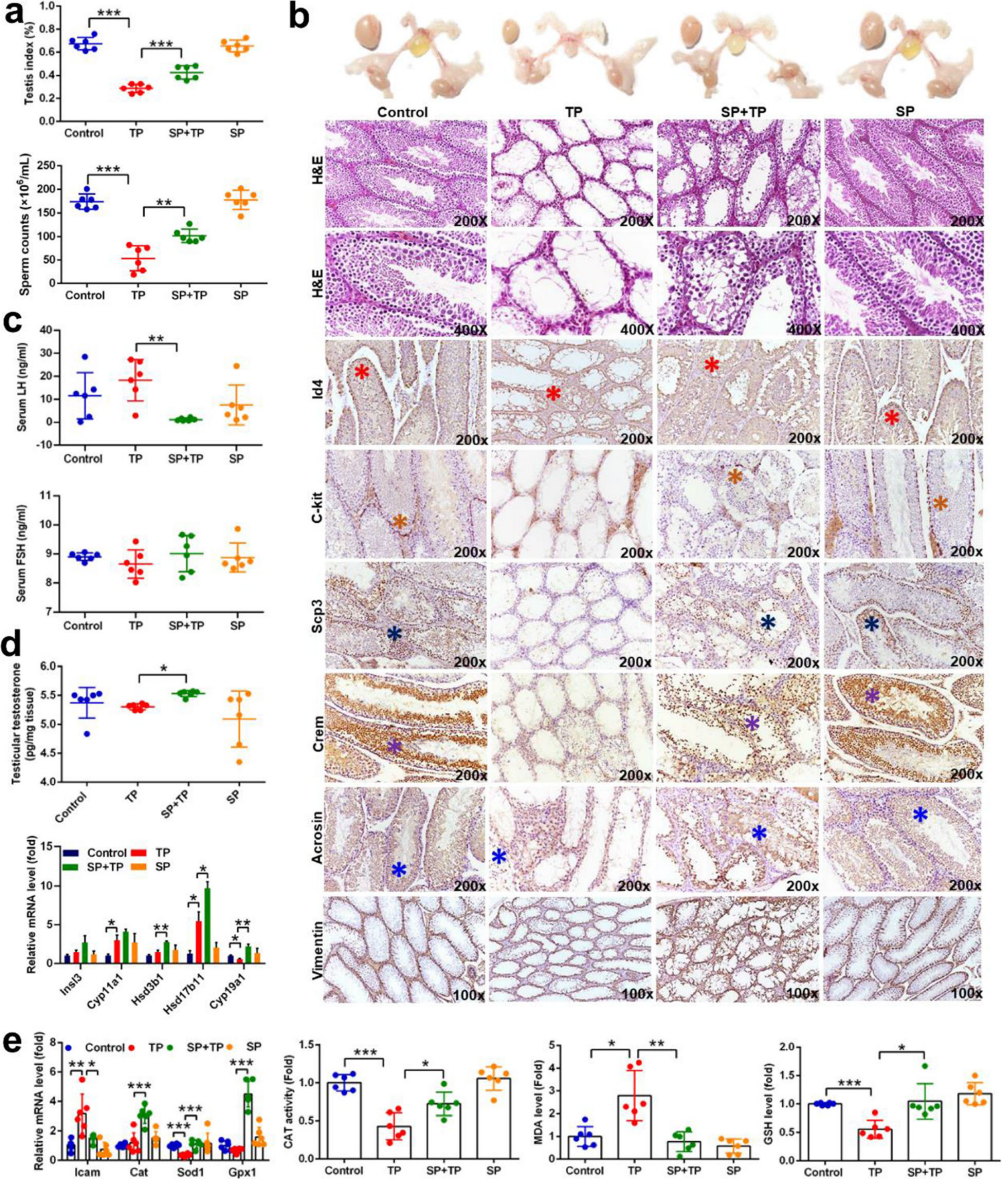
Spermine ameliorated testicular injury. **a** Testis index and sperm counts in epididymides. **b** Size and histology of testis (Id4 for spermatogonial stem cell; C-kit for differentiated spermatogonium; Scp3 for meiotic spermatocyte; Crem for late meiotic spermatocyte and round spermatid; Acrosin for haploid cell; Vimentin for sertoli cells). **c** Serum LH and FSH levels. **d** Testosterone levels and steroidogenic genes. **e** Inflammation factor and oxidative stress in testis. *P < 0.05, **P < 0.01, and ***P < 0.001 (*n* = 6)

Since polyamines were reported to play an essential role in reproductive processes and embryo/fetal development [26], we hypothesized that spermine might play a critical role in TP-induced testicular injury. Morphological analysis found that spermine increased the size of testis but did not influence epididymides, seminal vesicles, and the preputial gland (Fig. 3b). Spermine improved differentiated spermatogonium (marked by C-kit), meiotic spermatocyte (marked by Scp3), late meiotic spermatocytes (marked by Crem), round spermatid (marked by Crem and Acrosin), elongating spermatid (marked by Acrosin), and sertoli cell (marked by Vimentin) induced by TP (Fig. 3b). Testis indices and sperm counts in epididymides also supported the protective effect of spermine against testicular injury (Fig. 3a). Hormonal levels (luteinizing hormone (LH), follicle stimulating hormone (FSH), and testosterone) were not influenced by TP, while spermine could increase testosterone levels and testosterone- and androsterone-synthesis related gene mRNAs encoding the hydroxysteroid dehydrogenases HSD3B1 and HSD17B11. Spermine also reduced inflammation factor (*Icam* mRNA) and improved oxidative stress (*Cat*, *Sod1*, *Gpx1* mRNAs, CAT activity, malondialdehyde (MDA), and GSH levels). Furthermore, spermidine could also improve TP-induced testicular injury (Supplementary Fig. 5d).

**Fig. 4:**
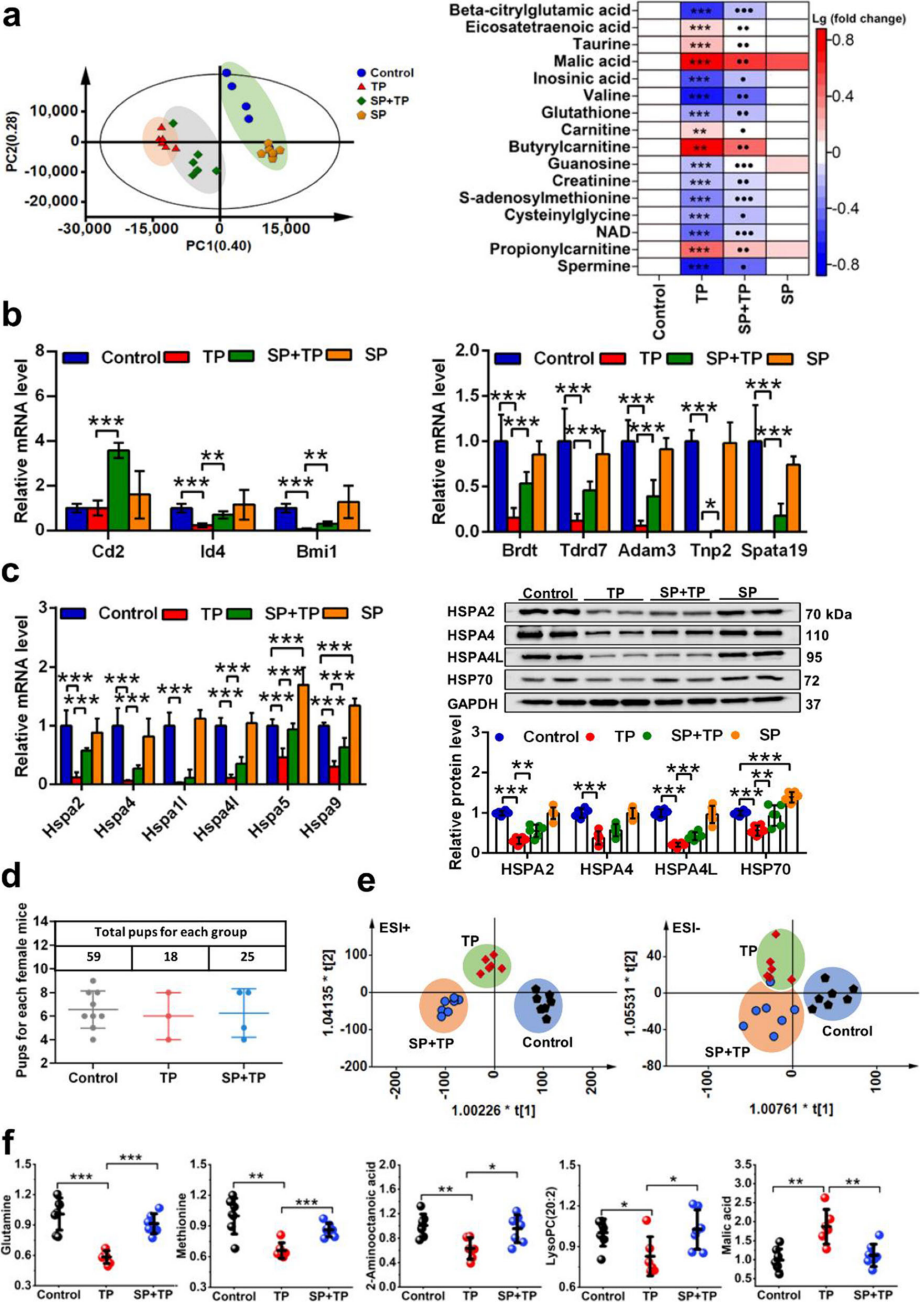
Spermine ameliorated metabolites and influenced the offspring. **a** PCA score plot for testis metabolome (integration of ESI+ and ESI−) and metabolites improved by spermine (control vs TP: **P < 0.01, and ***P < 0.001; TP vs SP+TP: ●*P* < 0.05, ●●*P* < 0.01, and ●●●*P* < 0.001; red represented the increase, while green represented the decrease, *n* = 6). **b** mRNA level of genes related to early and late spermatogenic events (*n* = 6). **c** mRNA and protein level of HSP70s (*n* = 6). **d** Total pups for each group and pups for each female mouse. **e** OPLS-DA score plot for plasma metabolome of the offspring (ESI+ and ESI− modes, *n* = 7). **f** Metabolites improved by spermine in plasma of the offspring (*n* = 7). *P < 0.05, **P < 0.01, and ***P < 0.001

Testicular metabolomics revealed that spermine administration to TP-exposed mice rendered them closer to the control group as revealed by the PCA score plot (Fig. 4a) as a result of significantly recovered metabolites, including glutathione, taurine, malic acid, carnitine, nicotinamide adenine dinucleotide (NAD+) and spermine in the testis (Fig. 4a). However, the concentration of spermine in testis, while statistically higher in the spermine + TP (SP + TP) group, was still lower than that in the vehicle-treated group, although spermine administration significantly increased N-acetylspermidine levels (Supplementary Figure 5a). In addition, mRNA levels of genes involved in early and late spermatogenic events and ATP utilization were also improved by spermine treatment (Fig. 4b and Supplementary Figure 5b). Moreover, spermine increased heat shock protein 70 family gene mRNA levels (heat shock protein family A member 2 (*Hspa2*), *Hspa4*, *Hspa4l*, *Hspa5*, and *Hspa9*) and protein levels (HSPA2, HSPA4L, and HSP70) after TP exposure, even under normal physiological conditions (Fig. 4c). The results indicated that increased HSP70s might be associated with the improvement of testicular dysfunction.

An intergenerational experiment was used to investigate the performance of offspring from male mice treated with TP and spermine. The results demonstrated that spermine increased the size of the litter reduced by TP (Fig. 4d) and normalized the plasma metabolome that was disrupted by TP (Fig. 4e). Four metabolites were upregulated, which were glutamine, methionine, 2-aminooctanoic acid, and LysoPC (20:2), and malic acid was downregulated (Fig. 4f). Organ indexes of heart, brain, testis, liver, kidney, and lung were not changed in the TP and SP + TP groups (Supplementary Figure 5c). These results suggest that spermine plays an important role in attenuating testicular dysfunction and increases the lower litter numbers found after TP treatment.

### HSP70s regulated by spermine protected against testicular injury

To reveal the potential mechanism of spermine in testicular toxicity in vitro, TM4 cells derived from sertoli cells, which are widely used for investigating testicular toxic mechanism of TP were employed [10, 27], and initially treated with spermine and TP for 24 h. Spermine remarkably reversed the TP-induced cytotoxicity as revealed by cell viability, lactate dehydrogenase (LDH) levels, MDA levels, ATP levels, expression of the mitochondrial-related mRNAs, and mitochondrial membrane potential (Supplementary Figure 6a–c). Spermine was found to play an important role in cell proliferation since eflornithine, an inhibitor to polyamine biosynthesis, profoundly restricted the growth of TM4 cells (Supplementary Figure 6d). The damage could be reversed by spermine supplementation (Supplementary Figure 6e). Furthermore, eflornithine potentiated TP-induced cytotoxicity (Supplementary Figure 6f).

**Fig. 5:**
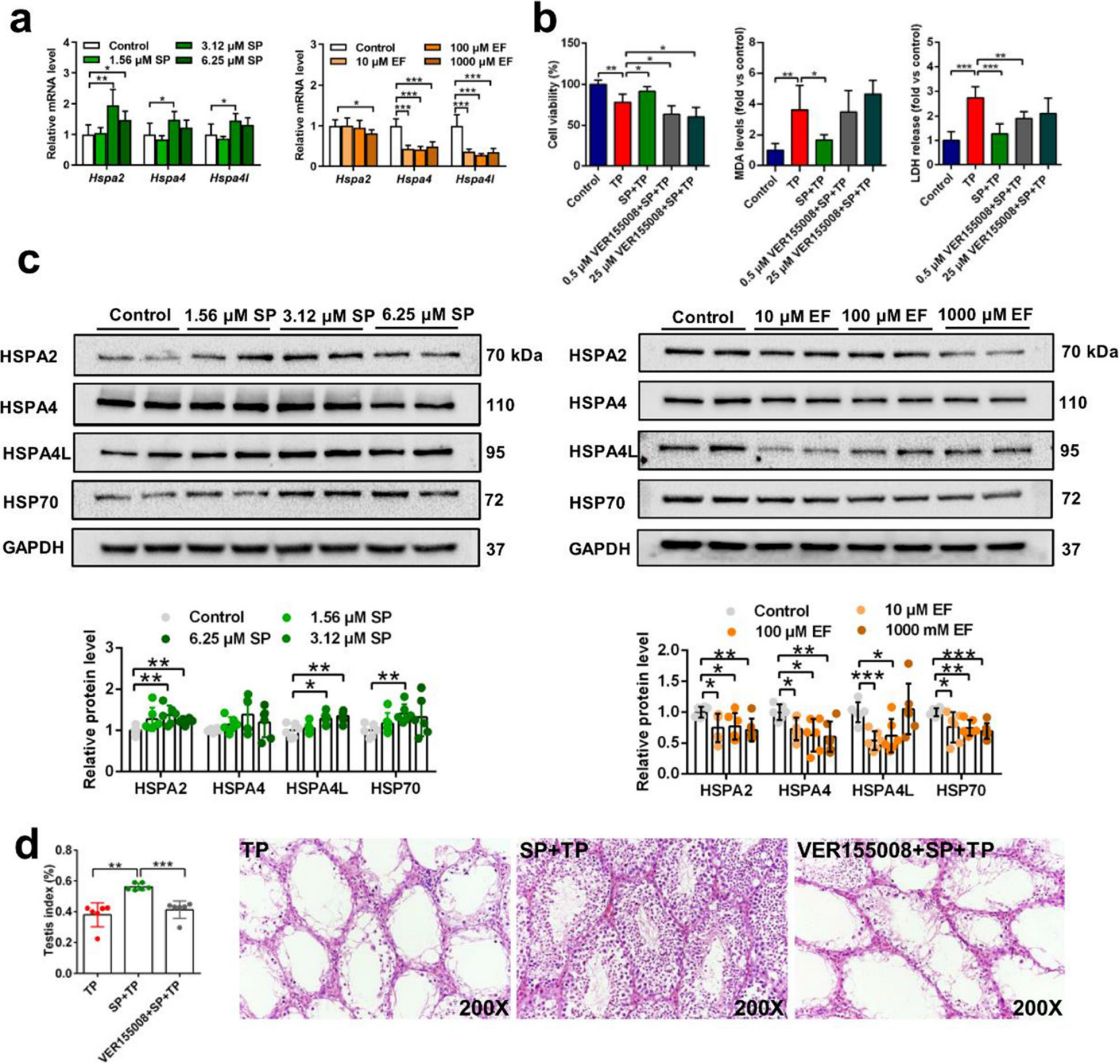
HSP70 regulated by spermine protected against testicular toxicity induced by TP in mice and TM4 cells. **a** mRNA levels of HSP70s after treatment with spermine or eflornithine, a polyamine pathway inhibitor. **b** The function of HSP70 was indicated by cell viability, MDA, and LDH levels after treated with a HSP70 inhibitor VER155008. The concentration of spermine was 6.25 μM. **c** Spermine increased protein levels of HSP70s and eflornithine decreased protein levels of HSP70s. **d** VER155008 reduced the protective effect of spermine in mice. EF: eflornithine. The concentration of TP used in this experiment was 80 nM in TM4 cell. *P < 0.05, **P < 0.01, and ***P < 0.001 (*n* = 6)

It was reported that levels of the polyamine exporter TPO1 in yeast was negatively correlated with the protein levels of HSPs [28], and HSPs played an important role in testicular toxicity in mice [29, 30]. Therefore, the relationship between HSPs and spermine was evaluated. Spermine could enhance and eflornithine could reduce the expression of HSP70s, including *Hspa2*, *Hspa4*, and *Hspa4l* mRNAs as well as HSP70, HSPA2, HSPA4, and HSPA4L proteins (Fig. 5a, c). More importantly, the protective effect of spermine was absent in TM4 cells and mice when VER155008, a HSP70s inhibitor, was introduced (Fig. 5b, d). These results showed that the protective effect of spermine on testicular injury was dependent on HSP70s.

### Loss of gut microbiota aggravated testicular injury

**Fig. 6:**
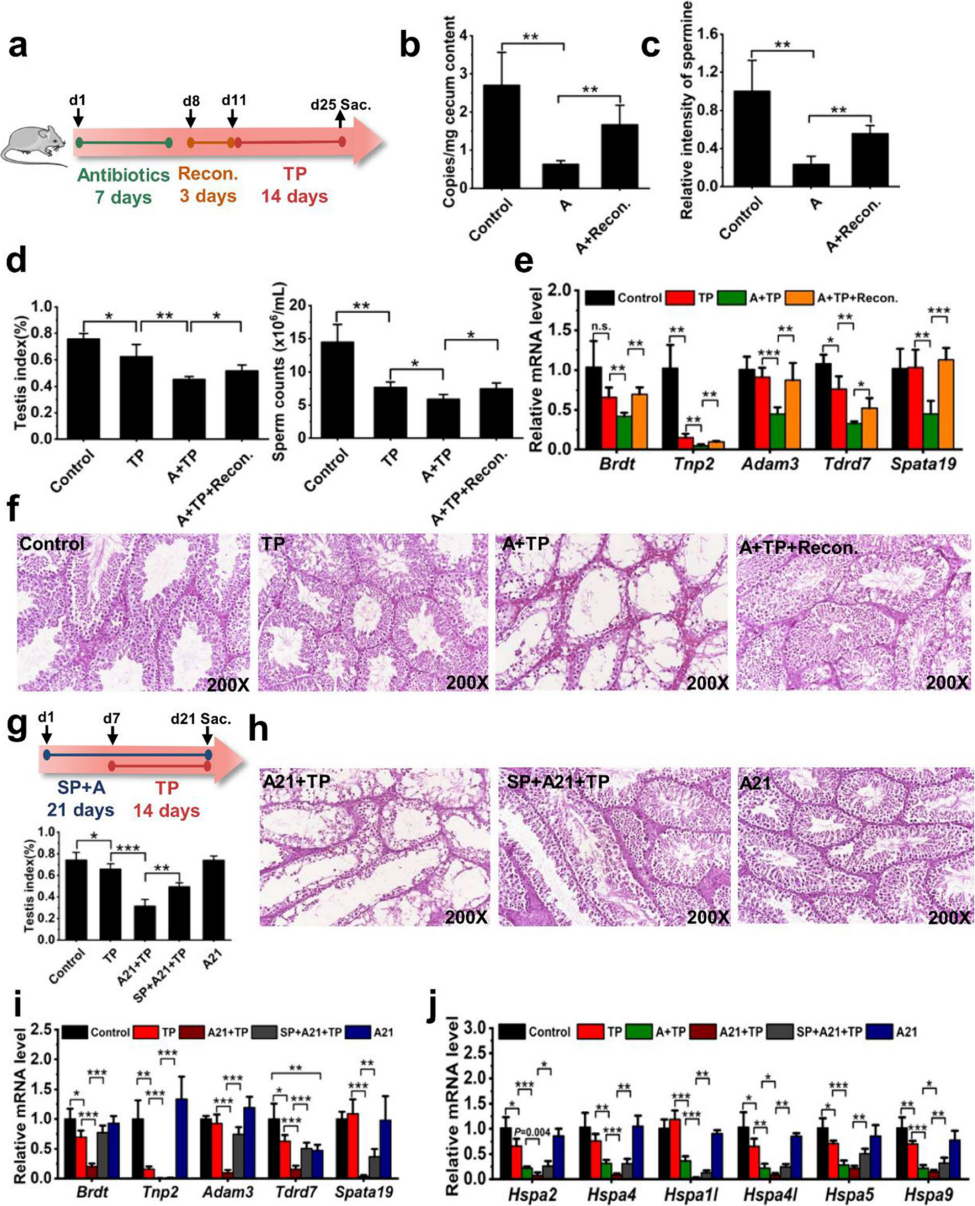
Loss of gut microbiota aggravated testicular injury. **a** Procedure to investigate the function of gut microbiota on TP-induced testicular injury. **b** Copies numbers of total bacteria in cecum content. **c** Polyamines levels in cecum content. **d** Testis index and sperm counts in epididymides. **e**, **i** mRNA level of genes related to spermatogenesis. **f**, **h** H&E staining of testis. **g** Procedure to validate the function of spermine derived from gut microbiota and testis index. **j** mRNA levels of genes encoding HSP70s. *P < 0.05, **P < 0.01, and ***P < 0.001 (*n* = 6). A: normal antibiotic treatment; Recon.: gut microbial transplantation; TP: triptolide treatment; A21: antibiotic treatment for 21 days

In order to determine the role of spermine and gut microbiota on testicular dysfunction, bacteria in the intestinal tract were depleted by antibiotics (ampicillin, neomycin, metronidazole, and vancomycin) and reconstructed by gut microbial transplantation (Fig. 6a). Total bacteria and spermine in the cecum lumen were decreased after antibiotics treatment, which were recovered by gut microbial reconstruction (Fig. 6b, c and Supplementary Figure 7a). The loss of gut microbiota potentiated TP-induced testicular toxicity as revealed by the improved testis index, sperm counts in epididymides, expression of genes involved in early and late spermatogenic events, H&E staining, hormonal levels, inflammation factors, and oxidative stress (Fig. 6d–f and Supplementary Figure 7b–e). These pathological manifestations could be improved following gut microbial transplantation from untreated mice (Fig. 6d–f and Supplementary Figure 7b–e).

Furthermore, supplementation with exogenous spermine elevated the testis index and attenuated the abnormal histopathological changes induced by antibiotics and TP (Fig. 6g, h). The expression of mRNAs encoded by genes related to spermatogenesis, ATP utilization, and HSP70s (*Hspa2*, *Hspa4*, *Hspa1l*, *Hspa4l*, *Hspa5*, and *Hspa9*) were increased by spermine intervention (Fig. 6i, j and Supplementary Figure 8). The abundance of gut microbiota was thus linked to testicular injury through spermine production.

### Testicular injury was improved by *Parabacteroides distasonis* transplantation

**Fig. 7:**
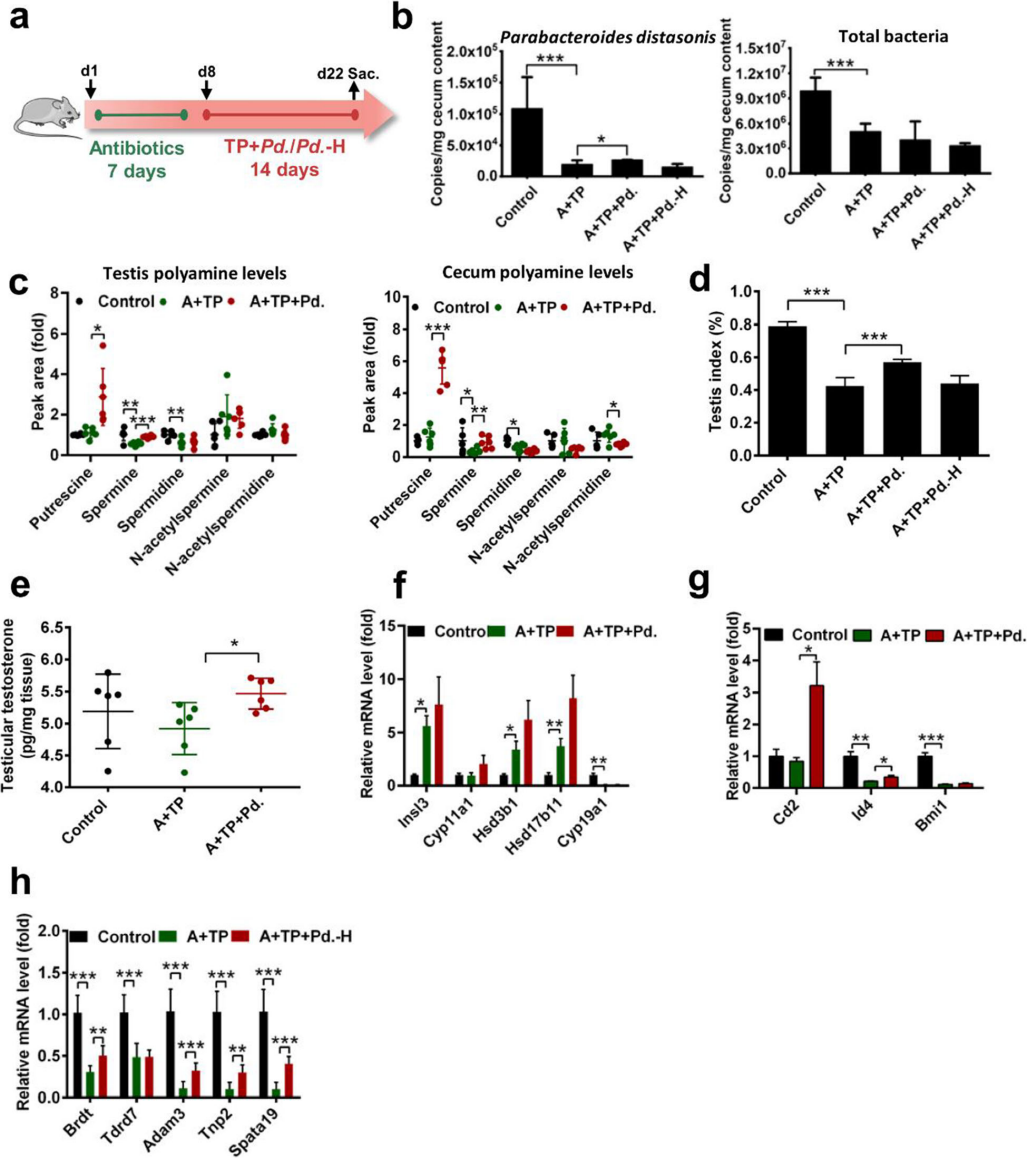
Testicular injury was improved by *Parabacteroides distasonis* transplantation. **a** Procedure for transplantation of *Parabacteroides distasonis*. **b** Copies of *Parabacteroides distasonis* and total bacteria in cecum content. **c** Testis and cecum polyamine levels. **d** Testis index. **e**, **f** Testosterone levels and steroidogenic genes. **g** mRNA level of genes related to early and late spermatogenic events. *P < 0.05, **P < 0.01, and ***P < 0.001 (*n* = 6). A: antibiotic; *Pd.*: *Parabacteroides distasonis*; *Pd*-H*.*: heat-killed *Parabacteroides distasonis*

To validate whether spermine derived from the gut microbiota was able to attenuate TP-induced testicular toxicity, *Parabacteroides distasonis,* a spermine-advantaged strain found to be decreased with testicular dysfunction, was transplanted to antibiotic-treated mice (Fig. 7a). As expected, *Parabacteroides distasonis* in the cecum lumen was significantly increased after transplantation, although the total number of bacteria was not significantly changed (Fig. 7b). *Parabacteroides distasonis* transplantation increased polyamine levels in testis and cecum as revealed by the increased spermine and putrescine levels (Fig. 7c). Improved histology, testis indices, testicular testosterone levels, expression of genes involved in early and late spermatogenic events, inflammatory factors and oxidative stress indicated that *Parabacteroides distasonis* could improve testicular damage caused by TP (Fig. 7d–h and Supplementary Figure 9). These results demonstrated that spermine-producing gut bacteria can protect testis from toxic exposures.

## Discussion

It is necessary to determine the harmful factors such as life styles and drugs that can influence human fertility in order to avoid the damage from toxic exposures, especially for individuals who are planning a pregnancy. In the present study, spermine deficiency in testis caused by both the host and gut microbiota was found to induce testicular injury in mice. Supplementation with exogenous spermine or transplantation with a bacterial strain favoring in spermine production reversed testicular dysfunction as revealed by histological injury, testis index, sperm counts, and downregulated mRNA levels of genes involved in early and late spermatogenic events, including *Cd2*, *Id4*, *Bmi1*, *Brdt*, *Tdrd7*, *Adam3*, *Tnp2*, and *Spata19* that are elevated upon TP exposure. *Cd2*, *Id4*, and *Bmi1* marks spermatogonial stem cells [31–33]. TP decreased *Id4* and *Bmi1* mRNAs, indicating that TP impairs spermatogenic stem cells, but surviving spermatogenic stem cells were also found by IHC which may imply the potential for reversibility of the TP effects.

In mammalian cells, spermine is produced initially by ODC, a rate-limiting enzyme in the biosynthesis pathway, followed by successive reactions of aminopropyl transfer via spermidine synthase (SRM) and spermine synthase (SMS). Conversely, spermine can be directly converted into spermidine without acetylation by SMOX or catalyzed by SAT to form N^1^-acetylspermine, which is easily transported by SLC3A2. On the other hand, spermine is transported by SLC22A16, an important transporter for polyamine uptake system, and eventually enters the metabolic cycle [34, 35]. The mRNAs levels of *Odc1* and *Slc22a16* were decreased by TP, indicating that the biosynthesis and uptake system of spermine were impaired. Further experiments showed that spermine derived from either supplementation or gut microbiota could ameliorate testicular dysfunction. However, the testicular injury did not rebound to normal levels following spermine supplementation, accompanied by the lower concentrations of spermine than the control group. Based on the above observation, we assumed that the effect of spermine on testis might be indirect which need further research in the future: spermine might be transferred from other tissues, such as the surrounding tissue, blood circulation, and cecum through several polyamine transport protein [36], which was stored in the form of putrescine, spermidine, and N-acetylspermidine in Supplementary Figure 5a. The increased polyamine levels in testis after spermine supplementation might come from endocytosis, although *Slc22a16* mRNA level was inhibited by TP [37]. These data demonstrated that spermine could protect against TP-induced testicular injury, suggesting a role for spermine in testicular function.

Spermine is present in many organisms including animals and some bacteria including *Actinobacteria*, *Firmicutes*, *Proteobacteria*, and *Bacteroidetes* [21]. As a positively charged amine ubiquitous in all organisms, spermine functions range from antioxidation, regulation of ion channels and bone development, inhibition of lipid synthesis, anabolic effects, maturation of the gut and immune system, and maintaining the normal physiology of reproduction [22]. The functions of polyamine synthesis genes (ODC, AMD, OAZ, AZIN, and SMS) on male reproductive system were evaluated using knock-out mice [38–44]. ODC and AMD were key enzymes in polyamine biosynthesis which were inhibited by TP. Previous study found ODC and ADM were correlated with the development of the spermatid and played an essential role for cell survival during early murine development [38–40]. Furthermore, OAZs and AZINs were modulators of ODC. TP inhibited *Odc1*, *Oaz1* and *3*, *Azin1*, and *2* mRNA levels, which confirmed previous data using other drugs that resulted in testicular toxicity like cyclophosphamide [41]. OAZ3 knock-out male mice were found to be infertile and produced aberrant spermatozoa [42]. AZIN2 knock-out male mice were fertile, but they decreased testicular putrescine and testosterone levels and the sperm motility [41]. Targeted disruption of SMS, a spermine synthesis gene, in embryonic stem cells failed to produce viable mice or led to neurological dysfunction, decreased body size, poor bone development, short life span, deafness, and sterility [43, 44]. All these previous studies showed that polyamine participated in testicular injury. In the present study, the spermine and polyamine-produced strain *Parabacteroides distasonis* was found to ameliorate testicular injury caused by TP, which could improve seminiferous tubules, counts of germ cells, and promote the expression genes involved in spermatogenesis. Furthermore, spermidine, the precursor of spermine, can also ameliorate testicular injury caused by TP in Supplementary Fig. 5d. Previous study reported that spermine and spermidine could show the similar effect in preventing bone loss and ameliorating aging-induced dementia [45, 46]. The increase of acetylated polyamines levels after TP treatment might majorly come from the increase of SSAT enzyme activity in Fig. 2b. On the other hand, the increased acetylpolyamine levels might come from the change of transfer through several polyamine transport protein families, including ATP-binging cassette transporters and protein potential-dependent solute carriers [36].

Gut microbiota-derived polyamines are a critical source for the polyamine pool of the host even though the uptake systems in organs are strictly regulated. Bacteria colonizing the intestinal tract produce polyamines mainly through transamination of the ingested amino acids by catalytic enzymes, particularly for arginine [23, 24]. Growing evidence demonstrated that supplementation with arginine and/or the probiotic strain *Bifidobacterium animalis subsp*. *lactis* LKM512 suppressed colonic cell senescence and prolonged life span dependent on the gut microbial polyamine production in mice [47, 48]. In our study, spermine and spermidine were decreased in cecum lumen after TP exposure, which may result from suppression of the gut microbiota involved in polyamine production. Perturbation of gut microbiota was identified as a destructive factor for testicular health evidenced by the antibiotics-enhanced testicular injury and bacterial-improved influence of *Parabacteroides distasonis* transplantation. These findings innovatively connected disrupted gut microbial metabolism and testicular dysfunction.

In this study, HSP70s regulated by spermine were demonstrated to protect against testicular injury. Treatment of TM4 cells with the HSP70s inhibitor VER155008 suppressed the growth of cells and increased cellular MDA and LDH levels. It was reported that levels of the polyamine exporter TPO1 in yeast was negatively correlated with the protein levels of HSPs, including HSP70s, HSP104s and HSP90s [28]. HSP70s plays an important role in maintaining the normal physiology of male reproduction. Targeted gene disruption of *Hsp2* resulted in impaired meiosis, germ cell apoptosis, and male infertility in mice [29]. An increased number of male mice deficient in *Hspa4* displayed impaired fertility, lower testis size, and anormal testicular histology [30]. In this study, the suppression of HSP70s in testis at least partially contributed to testicular injury, and spermine may exert its protective effect through upregulating the expression of the genes encoding HSP70s. Downregulation of HSP70s contributed to the therapeutic effects of TP on cancers since anti-cancer drugs targeting HSP70s were developed [49]. Thus, the double-edged sword effect of polyamines in life science seems to be associated with the expression of HSPs.

A pharmacologic approach used for male contraception remains a longstanding challenge in medicine. A previous study found that triptonide is a reversible non-hormonal male contraceptive agent in mice and non-human primates, and the male fertility recovered within 3–6 weeks following cessation of triptonide [50]. Triptonide appears to target junction plakoglobin and disrupts its interactions with SPEM1 during spermiogenesis, which contributed to its male infertility in mice and cynomolgus monkeys [50]. TP, an antispermatogenic agent, has a similar structure as triptonide. The reversibility of TP-induced testicular toxicity was also observed in our study (Fig. 1e). Therefore, studies on the role of TP in testicular function could be beneficial to develop male contraceptives in humans because of its reversibility.

## Conclusions

**Fig. 8:**
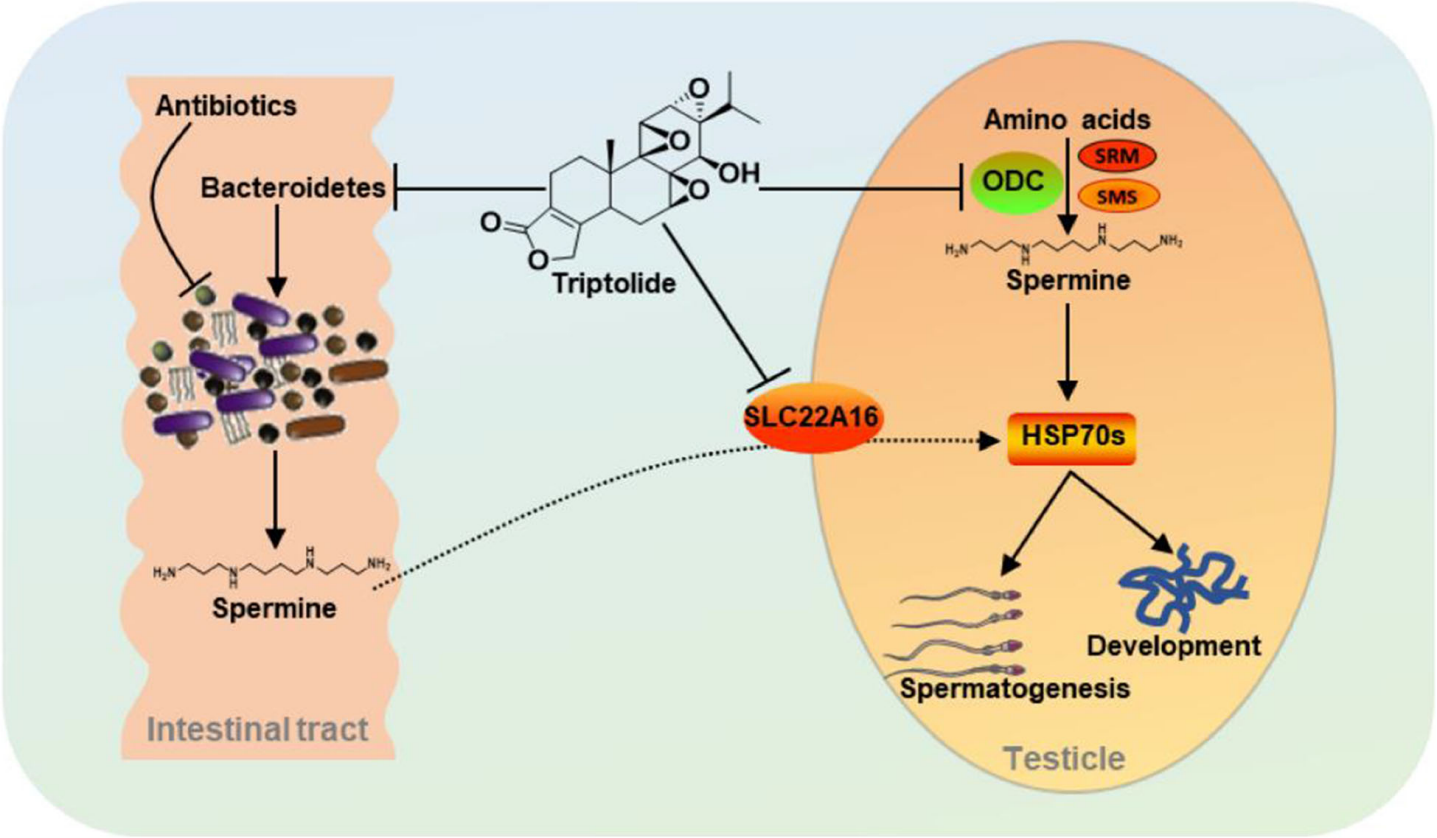
TP influenced gut microbiota and the function of testis. TP decreased the levels of ODC and SLC22A16 in testis, which impaired the biosynthesis of spermine and its uptake. ODC is responsible for the generation of putrescine from ornithine, which is further transformed into spermidine and spermine. *Parabacteroides distasonis* that produces spermine was decreased following TP exposure. The decrease of spermine concentration in testis resulted in the downregulation of HSP70s and testicular injury

In conclusion, spermine deficiency resulting from inhibition of polyamine biosynthesis and uptake in testis, and perturbation of the gut microbiota contributed to testicular dysfunction, which could be improved by spermine supplement or gut microbial transplantation. The protective effect of spermine and gut microbiota to mitigate TP-induced testicular injury largely depends on upregulation of HSP70s. The interaction between gut microbiota, TP, and testicular injury is shown in Fig. 8. The diversity and dynamics of gut microbiota could be considered as a strategy to prevent male reproductive disorders.

## Methods

### Animals

Specific pathogen-free (SPF) C57BL/6J mice (8–10 weeks old) were purchased from Liaoning Changsheng biotechnology Co., Ltd. (Liaoning, China), and maintained under a standard 12-h light/12-h dark cycle environment with free access to water and rodent chow. All animal experiments were approved by the West China Hospital, Sichuan University.

### TP treatment and spermine supplement

To study TP-induced testicular toxicity, reversibility, and time-dependent effects, mice were randomly assigned into two groups (*n* = 6): (1) control; (2) TP. TP group mice were intraperitoneally injected with TP (0.2 mg/kg, dissolved in 1% DMSO) daily for 14 days [51], and the dose of TP in clinics was 3.3 μg/kg in human (corresponding to 0.03 mg/kg in mice) [52]. Control mice were treated with 1% DMSO alone for 14 days. Part of the mice were killed at the 14th day to evaluate testicular toxicity (*n* = 6). After 14-day treatment, TP treatment was discontinued for 2 months to evaluate the reversibility (*n* = 6). To evaluate the time-dependent effect, 0, 2, 4, 6, 8, 10, 12, and 14 days testicular samples were collected after TP treatment (*n* = 6).

To further investigate the protective effect of spermine on TP-induced testicular injury, mice were randomly assigned to four groups (*n* = 12): (1) control; (2) TP; (3) SP+TP; (4) spermine (SP). The SP and SP + TP groups were treated with spermine-containing water (0.3 mM) for 17 days [45]. After spermine treatment for 3 days, the TP and SP+TP groups were intraperitoneally injected with TP (0.2 mg/kg) daily for 14 days. Twenty-four hours after the last dose of TP, mice (*n* = 6) were killed by CO_2_ asphyxiation and samples were collected. Another batch of male mice (*n* = 6) were matched with the normal female mice (1 male matching 2 female) for 3 months to study the growing performance of the offspring. Afterwards, body weights, numbers, organ indexes, and plasma metabolome profiles of the first litter (8 weeks old) were investigated (*n* = 7). Organ index was calculated by organ weight/body weight × 100% [53]. Plasma metabolome was carried out as previous study [54].

### Spermidine supplementation

To investigate the protective effect of spermidine on TP-induced testicular injury, mice were randomly assigned to two groups (*n* = 6): (1) TP; (2) SPD + TP. The SPD + TP group was treated with spermidine-containing water (0.3 mM) for 17 days [45]. After spermidine treatment for 3 days, the TP and SPD+TP groups were intraperitoneally injected with TP (0.2 mg/kg) daily for 14 days. Twenty-four hours after the last dose of TP, mice were killed by CO_2_ asphyxiation and samples were collected.

### Gut microbial depletion and transplantation

To determine the function of gut microbiota on TP-induced testicular injury, mice were randomly assigned to 6 groups (*n* = 6): (1) control; (2) TP; (3) antibiotics + TP (A + TP); (4) antibiotics + TP + microbial transplantation (A + TP + Recon.); (5) antibiotics (A); (6) antibiotics + microbial transplantation (A + Recon.). Antibiotics including ampicillin (0.25 mg/mL, Sigma), neomycin (0.25 mg/mL, Sigma), metronidazole (0.25 mg/mL, Sigma), and vancomycin (0.125 mg/mL, Sigma) were dissolved in autoclaved water and supplied ad libitum to eliminate gut microbiota of mice [51]. For microbial transplantation, fresh gut microbiota from the donor mice was prepared by suspending 50 mg cecum content in 1 mL sterilized PBS followed by centrifugation at 1000×*g*. The volume of microbial suspension was 200 μL/mouse [55]. A + TP, A + TP + Recon., A, and A + Recon. groups were treated with antibiotics for 7 days. After antibiotics treatment for 7 days, gut transplantation was conducted for 3 days starting from the 8th day in the A + TP + Recon. and A+Recon. groups. Starting from the 11th day, the TP, A + TP, and A + TP + Recon. groups were intraperitoneally injected with TP (0.1 mg/kg) daily for 14 days. Because antibiotics increased TP toxicity and caused some premature mouse death, a lower dose 0.1 mg/kg TP was used.

For validating the function of spermine on the gut microbiota and TP-induced testicular injury, mice were randomly assigned to 5 groups (*n* = 6): (1) control; (2) TP; (3) 21 days antibiotics + TP (A21 + TP); (4) spermine + 21 days antibiotics + TP (SP + A21 + TP); (5) 21 days antibiotics (A21). Spermine (0.3 mM) was co-dissolved with antibiotics in the drinking water and mice allowed free access for 21 days. After antibiotics treatment for 7 days, the TP, A21 + TP, and SP + A21 + TP groups were intraperitoneally injected with TP (0.1 mg/kg) daily for 14 days.

Microbial depletion was quantified by bacterial 16S rRNA copies using qPCR. *Parabacteroides distasonis* (ATCC8503) was used for standard curve construction by diluting DNA in a gradient from 10^4^ to 10^9^ copies/mL template. The DNA concentrations were measured by NanoDrop (Thermo Fisher Scientific, Germany). QPCR primers for eubacteria and *Parabacteroides distasonis* were listed in Supplementary Table 3.

### Parabacteroides distasonis transplantation

*Parabacteroides distasonis* was obtained from American Type Culture Collection (ATCC8503), which was cultured in brain-heart infusion medium (Huankai, China) at 37 °C in an anaerobic chamber for 24 h. Bacterial pellets were collected by centrifuging at 8000×*g* for 10 min at 4 °C. After resuspended in sterilized oxygen-free PBS, cultured bacterial cells were administrated orally to mouse at 0.2 mL containing 2 × 10^8^ CFU. The experimental groups were set as follows (*n* = 6): (1) control; (2) A+TP; (3) antibiotics+TP+*Parabacteroides distasonis* (A + TP + Pd.); (4) antibitoics + TP + heat-killed *Parabacteroides distasonis* (A + TP + Pd.-H). After antibiotics treatment for 7 days, A + TP, A + TP + Pd., and A + TP + Pd.-H groups were given *Parabacteroides distasonis* transplantation and TP treatment (0.1 mg/kg) daily for 14 days starting from the 8th day. Heat-killed *Parabacteroides distasonis* was treated parallelly.

### HSP70 inhibitor treatment

To investigate the effect of HSP70 on the protective effect of spermine, mice were randomly assigned to three groups (*n* = 6): (1) TP; (2) SP + TP; (3) VER155008 + SP + TP. TP, SP + TP, and VER155008 + SP + TP groups were given VER155008 (20 mg/kg, dissolved in 10% DMSO + 5% Tween80) [56], spermine-containing water (0.3 mM), and TP treatment (0.2 mg/kg) daily for 14 days. Testis, cecum contents, and plasma were collected 24 h after the last dose of TP.

### Untargeted metabolomics study and polyamine determination

To prepare the testis samples, 30 mg testis was extracted with 300 μL 50% acetonitrile containing 5 μM chlorpropamide as internal standard. After centrifugation at 18000×*g* for 20 min, the supernatant was mixed with 50% acetonitrile at a ratio of 1:1 followed by vortexing and centrifugation as above. Cecum content samples and the conditions for UPLC-QTOF-MS were prepared using a method described in a previous study [54].

Polyamine in cecum contents was extracted based as described in a previous study [57] and analyzed by UPLC-QTOF-MS. In brief, 50 mg of cecum content was mixed with 500 μL 5% perchloric acid solution without any previous derivatization followed by 20 min shaking and centrifugation at 18000×*g* for 20 min.

Raw data were processed with Agilent MassHunter Worksation Software and MetaboAnalyst 4.0 [58]. Mass Profinder Software (Agilent, USA) was utilized to generate a data matrix. SIMCA-P + 13.0 (Umetrics, USA) was applied to multivariate statistical analysis including PCA and orthogonal partial least squares-discriminant analysis (OPLS-DA). Altered metabolites were matched on the Human Metabolome Database. The chemical structures of the altered metabolites were identified by MS/MS spectrogram and authentic standard in Supplementary Tables 1 and 2. Pathway enrichment was conducted with MetaboAnalyst 4.0.

### ODC and SSAT activities and biochemical assessment

After 1 h incubation with TP at 37 °C, ODC and SSAT activities were measured according to previous studies [59, 60] and the detailed methods were provided in the Supplementary Information. CAT and MDA (Nanjing Jiancheng Bioengineering Institute, China), LDH (Cayman, USA), mitochondrial membrane potential (Beytime, Shanghai, China), and ATP (Beytime, China) were measured following the manufacturer’s instructions. GSH levels were measured by UPLC-QTOF-MS.

## Microbial genomic DNA extraction and high-throughput sequencing

Microbial genomic DNA in cecum content was extracted using the Stool Genomic DNA kit (CWBIO, China) according to the manufacturer's instructions. 16S rRNA was carried out with Illumina HiSeq platform, and the procedure of high-throughput sequencing and bioinformatics analysis was performed by Beijing Genomics Institute (Shenzhen, China).

### Semen evaluation, morphological, and histological examination

Epididymides were sheared with a surgical scissors in 600 μL PBS and incubated in 37 °C for 10 min to allow sperm to release. Subsequently, 10 μL stock solution was diluted to 1 mL with PBS and an optical microscopy-based hemocytometer was used to assess sperm concentrations. Hematoxylin and eosin (H&E) staining was carried as detailed in a previous report [54]. Morphological analysis of the male reproductive system included evaluation of testis, epididymides, seminal vesicles, and the preputial gland.

### QPCR, immunohistochemistry, ELISA, and western blot analyses

QPCR and western blot analyses were performed as detailed in a previous report [61]. Related primers were listed in Supplementary Table 3. Target mRNA levels were normalized to those of *18S*. The following antibodies were used: glyceraldehyde-3-phosphate dehydrogenase (GAPDH) (14C10, CST), HSP70 (4872, CST), HSPA2 (MAB6010, R&D Systems), HSPA4 (3303, CST,), HSPA4L (PA5-100822, ThermoFisher), Vimentin (ab92547, Abcam), Id4 (YT2272, Immunoway), C-kit (ab231780, Abcam), Scp3 (ab97672, Abcam), Crem (sc-390425, Santa Cruz), Acrosin (NBP2-14260, Novus). Serum LH (CEA441Mu, Cloud-Clone), serum FSH (CEA830Mu, Cloud-Clone), and testicular testosterone (582701, Cayman) were measured by ELISA kits.

### TM4 cells culture and treatment

TM4 cells were obtained from the ATCC (Manassas, VA, USA) and cultured in DMEM/F12 containing 2.5% fetal bovine serum and 5% horse serum. The drug concentration was selected as follows: 80 nM TP (corresponding to about 0.02 mg/kg TP in mice) [10]; 1.56 μM, 3.12 μM, and 6.25 μM spermine [45]; 10 μM, 100 μM, and 1000 μM eflornithine [62]; 0.5 μM and 25 μM VER155008 [63, 64]. TM4 cells were treated with drugs for 48 h in eflornithine-related experiments and treated for 24 h for other experiments. Cell viability was assessed using a method described in a previous study [10]. Total RNA was extracted from TM4 cells using TRIzol reagent (Lifetechnologies, USA) to measure gene expression.

### Statistics

Data analysis and visualization were performed by GraphPad Prism v.6 (GraphPad, USA) and OriginPro2018 (OriginLab, USA). A two-tailed student’s *t* test was applied to assess the significant difference between two groups, whereas one-way ANOVA followed by Tukey’s post hoc test was applied to multiple treatment comparison. Data were expressed as mean ± SD and *P* value less than 0.05 was considered as statistical significance. The sample size of mice (*n* = 6) was chosen based on previous animal studies [65, 66].

## Supplementary Information

**Additional file 1: Supplementary Table 1.** Metabolites altered by TP in testis.

**Additional file 2: Supplementary Table 2.** Metabolites altered by TP in cecum contents.

**Additional file 3: Supplementary Table 3.** Overview of primer sequences.

**Additional file 4: Supplementary Figure 1.** Various metabolites and pathways were influenced by TP. a Body weight after TP treatment. b VIP value and significant difference of the changed metabolites in testis. c Pathway enrichment of the metabolites disrupted by TP.

**Additional file 5: Supplementary Figure 2.** Time-dependent effects of TP-induced testicular toxicity. Id4 for spermatogonial stem cell; C-kit for differentiated spermatogonium; Scp3 for meiotic spermatocyte; Crem for late meiotic spermatocyte and round spermatid; Acrosin for haploid cell; Vimentin for sertoli cell.

**Additional file 6: Supplementary Figure 3.** Polyamine pathway was influenced by TP. a mRNA levels of genes involved in spermine synthesis, metabolism and transport. b Spectrograms of spermine and spermidine. c Correlation between the testis index and levels of spermine and spermidine in testis. d Polyamine levels. *P<0.05, **P<0.01, and ***P<0.001.

**Additional file 7: Supplementary Figure 4.** High-throughput sequencing results of mice treated with TP. a Relative abundance of bacterial class, order, family, genus, and species in cecum content based on 16S rRNA sequencing. b Relative abundance of parabacteroides in genus.

**Additional file 8: Supplementary Figure 5.** The protective effect of spermine on TP-induced testicular dysfunction. a Relative intensity of polyamines in testis after supplement with exogenous spermine. b mRNA level of genes related to mitochondrial function and ATP utilization. c Body weight and tissue index (tissue weight/body weight × 100%) of the offspring. d The protective effect of spermidine on TP-induced testicular dysfunction. *P<0.05, **P<0.01, and ***P<0.001.

**Additional file 9: Supplementary Figure 6.** Spermine protected TP-induced testicular injury in TM4 cells. a The protective effect of spermine was evaluated by cell viability, extracellular LDH levels, and MDA concentrations in cells. b Mitochondrial injury was assessed by cellular ATP levels and expression of the mitochondrial-related genes *Sdhb*, *Tfam*, and *Uqcrc1*. c Representative photographs and quantitative analysis of mitochondrial membrane potential. d Eflornithine inhibited cell proliferation. e Spermine reversed the inhibited cell proliferation by eflornithine. The concentration of spermine was 6.25 μM. f Eflornithine aggravated TP-induced testicular injury. *P<0.05, **P<0.01, and ***P<0.001.

**Additional file 10: Supplementary Figure 7.** Loss of gut microbiota aggravated testicular injury. a Testicular polyamine levels. b Serum LH and FSH levels. c Testosterone level and steroidogenic genes. d Spermatogonial stem cell-related genes. e Inflammatory factor and oxidative stress. *P<0.05, **P<0.01, and ***P<0.001.

**Additional file 11: Supplementary Figure 8.** mRNA level of genes related to mitochondrial function and ATP utilization. *P<0.05, **P<0.01, and ***P<0.001.

**Additional file 12: Supplementary Figure 9.** *Parabacteroides distasonis* protects TP-induced testicular injury. a H&E staining. b Inflammatory factor and oxidative stress. A: antibiotic; TP: triptolide; *Pd.*: *Parabacteroides distasonis*; *Pd*-H*.*: heat-killed *Parabacteroides distasonis*. *P<0.05, **P<0.01, and ***P<0.001.

**Additional file 13: Supplementary Methods (DOCX 18 kb)**

## Data Availability

Data that support the findings detailed in this study are available in the supplementary information and this article. Any other source data perceived as pertinent are available, on reasonable request, from the corresponding author.

## Abbreviations

AMD: Adenosylmethionine decarboxylase
AZIN: Antizyme inhibitor
BMI1: B lymphoma Mo-MLV insertion region 1
CREM: Cyclic AMP-responsive element modulator
FSH: Follicle stimulating hormone
HSP: Heat shock protein
ID4: Inhibitor of differentiation 4
IHC: Immunohistochemistry
LH: Luteinizing hormone
MDA: Malondialdehyde
OAZ: Ornithine decarboxylase antizyme
ODC: Ornithine decarboxylase
PCA: Principal component analysis
SCP3: Synaptonemal complex protein 3
SLC22A16: Solute carrier family 22 member 16
SMS: Spermine synthase
SSAT: Spermidine/spermine-N^1^-acetyltransferase
TP: Triptolide
