# Supplementary material for: Polyamine metabolism links gut microbiota and testicular dysfunction
Source: Microbiome. 2021 Nov 11;9:224. doi: 10.1186/s40168-021-01157-z (PMC8582214; doi:10.1186/s40168-021-01157-z)
Supplement: Supplementary file 2 — Additional file 1: Supplementary Table 1. Metabolites altered by TP in testis. [file 40168_2021_1157_MOESM2_ESM.docx]

**Supplementary** **Table 1. Metabolites altered by TP in testis**

| Metabolites | Mass | RT (min) | | Modes | Main fragments | ppm  error |
| --- | --- | --- | --- | --- | --- | --- |
|  |  | RP | HILIC |  |  |  |
| Propionylcarnitine | 217.130 | 1.01 |  | ESI^+^ | 57; 85; 100 | 6.5 |
| LysoPC(22:6) | 567.333 | 10.42 |  | ESI^+^ | 86; 104; 184; 347; 437 | -0.9 |
| NAD | 664.117 | 1.01 |  | ESI^+^ | 152; 212; 328; 346; 409 | -0.1 |
| LysoPE(22:6) | 525.289 | 10.33 |  | ESI^+^ | 62; 177; 385; 465; 508 | -6.1 |
| steinylglycine | 178.040 | 1.00 | 5.12 | ESI^+^ | 119; 136; 147 | 6.8 |
| LysoPE(18:1) | 479.305 | 11.09 |  | ESI^+^ | 62; 155; 339; 462 | -8.0 |
| Pantothenic acid | 219.110 | 3.67 |  | ESI^-^ | 146; 88 | 3.1 |
| S-adenosylmethionine* | 399.149 | 1.00 | 6.52 | ESI^+^ | 90; 132; 136; 282 | -9.9 |
| LysoPE(16:0) | 453.287 | 10.76 |  | ESI^+^ | 62; 313; 393; 436 | -3.2 |
| Alpha-aminoenanthic acid | 145.110 | 0.88 | 5.44 | ESI^+^ | 60; 72; 84; 87; 102 | 1.9 |
| 2-Methylbutyroylcarnitine | 245.162 | 4.27 |  | ESI^+^ | 85; 57; 60 | 2.9 |
| Sphingosine | 299.282 | 9.23 |  | ESI^+^ | 55; 57; 88; 263 | 1.4 |
| Pyroglutamate | 129.043 | 0.97 |  | ESI^+^ | 56; 72; 84 | -3.2 |
| Creatinine* | 113.059 | 0.85 |  | ESI^+^ | 56; 58; 84 | -0.8 |
| LysoPE(20:4) | 501.286 | 10.35 |  | ESI^+^ | 62; 95; 361; 459; 485 | -0.9 |
| Spermine* | 202.215 | 0.76 |  | ESI^+^ | 129; 112; 84; 58 | 3.7 |
| Glutamic acid* | 147.052 | 0.93 |  | ESI^+^ | 56; 84; 102; 130 | 7.9 |
| Guanosine | 283.092 | 1.01 |  | ESI^+^ | 80; 110; 135; 152 | -1.2 |
| Indoleacrylic acid | 187.063 | 3.72 |  | ESI^+^ | 91; 115; 126; 143; 170 | 1.8 |
| LysoPC(18:2) | 519.333 | 10.45 |  | ESI^+^ | 184; 104; 86; 124; 60 | -1.0 |
| Docosapentaenoic acid | 330.254 | 14.2 |  | ESI^-^ | 59; 165; 231; 285 | 5.7 |
| Arginine* | 174.111 | 0.81 | 5.89 | ESI^+^ | 58; 60; 70; 72; 116 | 3.9 |
| Phenylalanine* | 165.078 | 1.03 |  | ESI^+^ | 93; 120; 103 | 5.9 |
| Methionine* | 149.050 | 1.00 | 4.87 | ESI^+^ | 56; 61; 87; 104; 133 | 7.0 |
| Inosinic acid | 348.045 | 1.00 | 4.91 | ESI^-^ | 78; 96; 135; 143; 296 | 6.0 |
| Alanine* | 89.048 | 0.88 | 5.36 | ESI^+^ | 56; 72 | -3.6 |
| LysoPC(20:4) | 543.333 | 10.45 |  | ESI^+^ | 60; 104; 166; 184; 526 | -0.9 |
| Butyrylcarnitine | 231.147 | 3.45 |  | ESI^+^ | 60; 85; 144; 173 | 0.3 |
| Proline* | 115.064 | 0.90 | 5.29 | ESI^+^ | 58; 59; 70; 74 | -5.8 |
| Glutamine* | 146.070 | 0.92 | 5.3 | ESI^+^ | 56; 84 | -5.9 |
| Tryptophan* | 204.090 | 3.71 |  | ESI^+^ | 91; 118;130; 143; 170 | -0.6 |
| 2-Aminooctanoic acid | 159.125 | 0.93 | 5.24 | ESI^+^ | 58; 60; 72; 85; 102 | 5.8 |
| L-Acetylcarnitine* | 203.117 | 0.98 | 5.28 | ESI^+^ | 85; 145; 60 | -6.1 |
| L-Carnitine* | 161.105 | 0.84 | 5.41 | ESI^+^ | 60; 85; 102; 103 | 1.2 |
| LysoPC(18:1) | 521.350 | 11.25 |  | ESI^+^ | 60; 86; 104; 184; 504 | -3.6 |
| Malic acid* | 134.021 | 0.93 |  | ESI^-^ | 71; 115 | 3.9 |
| Hypoxanthine | 136.039 | 1.00 | 2.34 | ESI^+^ | 55; 82; 94; 110; 119 | -3.6 |
| LysoPC(16:0) | 495.334 | 10.91 |  | ESI^+^ | 60; 86; 104; 124; 184 | -3.1 |
| Arabinosylhypoxanthine | 268.082 | 1.02 |  | ESI^+^ | 55; 61; 110; 119; 137 | -4.6 |
| Taurine* | 125.014 | 0.81 | 4.12 | ESI^-^ | 79; 94; 106 | 5.3 |
| LysoPC(18:0) | 523.368 | 12.34 |  | ESI^+^ | 60; 104; 184; 341; 506 | -8.0 |
| Glutathione* | 307.083 | 1.01 |  | ESI^+^ | 76; 84; 116; 144; 162 | 2.6 |
| DHA | 328.238 | 13.63 |  | ESI^-^ | 59; 149; 163; 229; 283 | 6.8 |
| Eicosatetraenoic acid | 304.239 | 13.83 |  | ESI^-^ | 59; 83; 183; 259; 285 | 4.0 |
| Glutathione* | 307.082 | 1.01 | 5.08 | ESI^-^ | 128; 143; 160; 254; 272 | 5.9 |
| Beta-citrylglutamic acid | 321.067 | 1.01 | 4.38 | ESI^-^ | 86; 128; 216; 284; 302 | 8.1 |
| Adenosine* | 267.098 | 1.00 | 3.38 | ESI^+^ | 57; 94; 110; 119; 136 | -4.7 |
| Valine* | 117.079 | 0.88 | 5.67 | ESI^+^ | 55; 58; 72; 87; 102 | -0.2 |
| Creatine* | 131.069 | 0.88 | 5.37 | ESI^+^ | 58; 72; 87; 90; 114 | 3.6 |
| Spermidine* | 145.158 | 0.66 |  | ESI^+^ | 72; 84; 112; 129 | 0.3 |
| Putrescine* | 88.100 | 0.65 |  | ESI^+^ | 55; 72 | 1.5 |
| N-acetylspermine | 244.226 | 0.66 |  | ESI^+^ | 100; 129; 171 | 1.6 |
| N-acetylspermidine | 187.168 | 0.71 |  | ESI^+^ | 100; 171 | 0.5 |
| GSH* | 307.084 | 0.89 |  | ESI^+^ | 76; 84; 116;130; 162 | -1.2 |

*Metabolites identified by authentic standards.
