# Supplementary material for: Polyamine metabolism links gut microbiota and testicular dysfunction
Source: Microbiome. 2021 Nov 11;9:224. doi: 10.1186/s40168-021-01157-z (PMC8582214; doi:10.1186/s40168-021-01157-z)
Supplement: Supplementary file 3 — Additional file 2: Supplementary Table 2. Metabolites altered by TP in cecum contents. [file 40168_2021_1157_MOESM3_ESM.docx]

**Supplementary** **Table 2. Metabolites altered by TP in cecum content.**

| Metabolites | Mass | RT (min) | | Modes | Main fragments | ppm  error |
| --- | --- | --- | --- | --- | --- | --- |
|  |  | RP | HILIC |  |  |  |
| Aspartic acid* | 133.038 | 0.86 | 5.07 | ESI^-^ | 71; 88; 115 | -3.7 |
| Arginine* | 174.112 | 0.86 | 5.86 | ESI^+^ | 58; 60; 70; 72; 116 | -1.9 |
| Proline* | 115.063 | 0.89 | 5.29 | ESI^+^ | 58.065; 59.072; 70.065; 74.060 | 2.9 |
| Phenylalanine* | 165.078 | 2.71 |  | ESI^+^ | 77; 93; 103; 120; 131;150 | 5.9 |
| Lysopine | 218.126 | 2.00 |  | ESI^+^ | 72; 84; 86; 132; 173; 201 | 3.0 |
| o-Tyrosine | 181.074 | 1.01 |  | ESI^+^ | 74; 91; 119; 123; 136; 147; 165 | -0.6 |
| Pyroglutamate* | 129.044 | 1.02 |  | ESI^-^ | 56; 72; 84 | -8.9 |
| 2-Hydroxyglutaric acid | 148.038 | 1.02 |  | ESI^-^ | 129; 101; 89; 85; 59 | -5.6 |
| 2-Aminonicotinic acid | 138.044 | 0.98 | 5.24 | ESI^+^ | 66; 68; 81; 93; 95; 121 | -7.8 |
| Leu-Val-Ser | 317.193 | 4.44 |  | ESI^+^ | 72; 86; 113; 143; 159; 173; 205; 257 | 2.2 |
| Phenylalanyl-isoleucine | 278.163 | 4.71 |  | ESI^+^ | 77; 86; 120; 136; 143; 159; 185; 210; 233 | 0.2 |
| Valyl-valine | 216.147 | 1.76 |  | ESI^+^ | 55; 72; 84; 131 | 1.8 |
| Taurocholic acid* | 515.295 | 7.33 |  | ESI^-^ | 79; 124 | -6.5 |
| Cholic acid* | 408.289 | 8.74 |  | ESI^-^ | 289; 325; 343; 389 | -3.5 |
| Lithocholic acid* | 376.300 | 12.11 |  | ESI^-^ | 339; 356 | -6.0 |
| I-Urobilinogen | 592.328 | 6.37 |  | ESI^-^ | 547; 504; 423; 292; 245; 120 | -3.2 |
| LysoPE(16:0) | 453.288 | 10.88 |  | ESI^-^ | 255; 196; 152; 366;214 | -5.4 |
| LysoPE(14:0) | 425.257 | 9.69 |  | ESI^-^ | 227; 196; 209; 78; 140 | -6.5 |
| LysoPE(15:0) | 439.273 | 10.09 |  | ESI^-^ | 140; 196; 241 | -7.1 |
| Succinic acid* | 118.026 | 1.64 | 4.90 | ESI^-^ | 55; 73; 99 | 5.2 |
| Oxypurinol | 152.034 | 1.02 |  | ESI^-^ | 108; 80; 65 | -3.8 |
| Creatine* | 131.070 | 0.89 | 5.37 | ESI^+^ | 58; 72; 87; 90; 114 | -4.0 |
| Linoleamide | 279.256 | 13.05 |  | ESI^+^ | 97; 111; 264 | 0.8 |
| Cervonoyl ethanolamide | 372.266 | 7.62 |  | ESI^+^ | 81; 107; 135; 159; 173; 235; 273; 319; 337; 355 | 1.2 |
| Tetracosahexaenoic acid | 356.272 | 8.94 |  | ESI^+^ | 93; 107; 135; 149; 187; 207; 221; 229; 247; 261 | -1.3 |

*Metabolites identified by authentic standards.
