# Supplementary material for: Polyamine metabolism links gut microbiota and testicular dysfunction
Source: Microbiome. 2021 Nov 11;9:224. doi: 10.1186/s40168-021-01157-z (PMC8582214; doi:10.1186/s40168-021-01157-z)
Supplement: Supplementary file 4 — Additional file 3: Supplementary Table 3. Overview of primer sequences. [file 40168_2021_1157_MOESM4_ESM.docx]

**Supplementary** **Table 3. Overview of primer sequences**

| **Genes** | **Forward primer** | **Reverse primer** |
| --- | --- | --- |
| *Amd1* | TTTCCCAAATGGAGCAGCA | GCTGGGTCAAGCTCACTCATC |
| *Oaz1* | GAGTTCGCAGAGGAGCAACT | CCAAGAAAGCTGAAGGTTCG |
| *Oaz2* | AGTAAGTGTCCCCAGCTCCA | ATCTTCGACAGTGGGTGAGG |
| *Azin1* | CTTTCCACGAACCATCTGCT | TTCCAGCATCTTGCATCTCA |
| *Azin2* | GCTTAGAGGGAGCCAAAGTG | CTCAGCAAGGATGTCCACAC |
| *Insl3* | CAGTGCCGCCACCAACGCTGT | AGGTTCATGATGGGGCTTCTTGG |
| *Cyp11a1* | AAGTATGGCCCCATTTACAGG | TGGGGTCCACGATGTAAACT |
| *Hsd3b1* | GTTGTCATCCACACTGCTGC | AAGATGAAGGCTGGCACACT |
| *Hsd17b11* | AAGAACGGCATCGAGGAAACA | TCCTGCGTAGCAAATAAGTCTG |
| *Cyp19a1* | TCAAGGGCGAGATGATAAGG | CGCTATTTGGCCTCAGAAGA |
| *Cd2* | GCCACCTGGATGCTAGAGG | GGCTTCACAGAGTTGTCGG |
| *Id4* | TGAACAAGCAGGGTGACAG | CGGTGGCTTGTTTCTCTTAATTTC |
| *Bmi1* | AAACCAGACCACTCCTGAACA | TCTTCTTCTCTTCATCTCATTTTTGA |
| *Icam* | AACAGTTCACCTGCACGGAC | GTCACCGTTGTGATCCCTG |
| *Cat* | CGACCAGGGCATCAAAAACTT | AACGTCCAGGACGGGTAATTG |
| *Sod1* | AAGCGGTGAACCAGTTGTGTT | AGCCTTGTGTATTGTCCCCATACT |
| *Gpx1* | TGGACTGGTGGTGCTCG | CGTCACTGGGTGTTGGC |
| *Brdt* | AGGGAAGCCAGTGAAAGCAT | TCCTTCGCATTATGCTCCAG |
| *Spata19* | TGTCCAAAGAGTGTCCACCTCA | CAAGGGCTCAGCGTTTAGAGT |
| *Tdrd7* | CTAAGGGCTGTCCTGCAGTC | TGAGAGTTGCCTTTGGCTTT |
| *Tnp2* | GTAGCTCAGGGCGAAGATACAA | TTCCTGTGACATCATCCCAAC |
| *Adam3* | GGTAACGACAGCCAGCAGTAAT | GCTTCTTGGTTGTGGTCTTCTT |
| *Mat2b* | AGGGAACCTTTCACTGGTCTG | ATTTGGAGCAATCGAGCTGAG |
| *Sms* | CACAGCACGCTCGACTTCAA | TGCCATTCTTGTTCGTGTAAGTT |
| *Slc3a2* | ACCTACTGAACACTCCACCG | TCATGTCCACTTCGGTGTCC |
| *Srm* | ACATCCTCGTCTTCCGCAGTA | GGCAGGTTGGCGATCATCT |
| *Odc1* | GACGAGTTTGACTGCCACATC | CGCAACATAGAACGCATCCTT |
| *Sat1* | GAGAACACCCCTTCTACCACT | GCCTCTGTAATCACTCATCACGA |
| *Sat2* | AGGTTGCCCTGAATAAGGGTT | CTCGCCTTCAAATCGAAAGGA |
| *Smox* | GACAGTGCGGATGACCCTC | GATAGATAGGATTCCCGTGGGAT |
| *Poax* | CTTCGGTGGTGTAGTGGAGC | TCCGATAATTCTTTCTCCCCCAG |
| *Oaz3* | CCACAGCCAGCTTAAAGAACT | CTTTCTCGGTTGCCTTGATCC |
| *Slc22a16* | GGGAACTCACGAGATGTAGCA | GTAGCCATCGAAGCAAGGACT |
| *Atp1a4* | TCAGGAGTCTGTTCCCATAGCTAA | GGAGAGCTGACTCGGAAGCA |
| *Sdhb* | AATTTGCCATTTACCGATGGGA | AGCATCCAACACCATAGGTCC |
| *Tfam* | ATTCCGAAGTGTTTTTCCAGCA | TCTGAAAGTTTTGCATCTGGGT |
| *Uqcrc1* | AGACCCAGGTCAGCATCTTG | GCCGATTCTTTGTTCCCTTGA |
| *Hspa2* | GCGTGGGGGTATTCCAACAT | TGAGACGCTCGGTGTCAGT |
| *Hspa4* | TTCCTCAACTGCTACATCGCT | CCTGTCGCTGTACTCGTTGG |
| *Hspa1l* | TCACGGTGCCAGCCTATTTC | CGTGGGCTCATTGATTATTCTCA |
| *Hspa4l* | TTCCTCAACTGCTACATCGCT | CCTGTCGCTGTACTCGTTGG |
| *Hspa5* | ACTTGGGGACCACCTATTCCT | ATCGCCAATCAGACGCTCC |
| *Hspa9* | ATGGCTGGAATGGCCTTAGC | ACCCAAATCAATACCAACCACTG |
| Eubacteria | ACTCCTACGGGAGGCAGCAGT | ATTACCGCGGCTGCTGGC |
| *Parabacteroides distasonis* | GGACACGTCCCGCACTTTAT | TTCTGAGAGGAAGGTCCCCC |
| *18S* | ATTGGAGCTGGAATTACCGC | CGGCTACCACATCCAAGGAA |
