# Supplementary material for: Polyamine metabolism links gut microbiota and testicular dysfunction
Source: Microbiome. 2021 Nov 11;9:224. doi: 10.1186/s40168-021-01157-z (PMC8582214; doi:10.1186/s40168-021-01157-z)
Supplement: Supplementary file 5 — Additional file 4: Supplementary Figure 1. Various metabolites and pathways were influenced by TP. a Body weight after TP treatment. b VIP value and significant difference of the changed metabolites in testis. c Pathway enrichment of the metabolites disrupted by TP. [file 40168_2021_1157_MOESM5_ESM.docx]

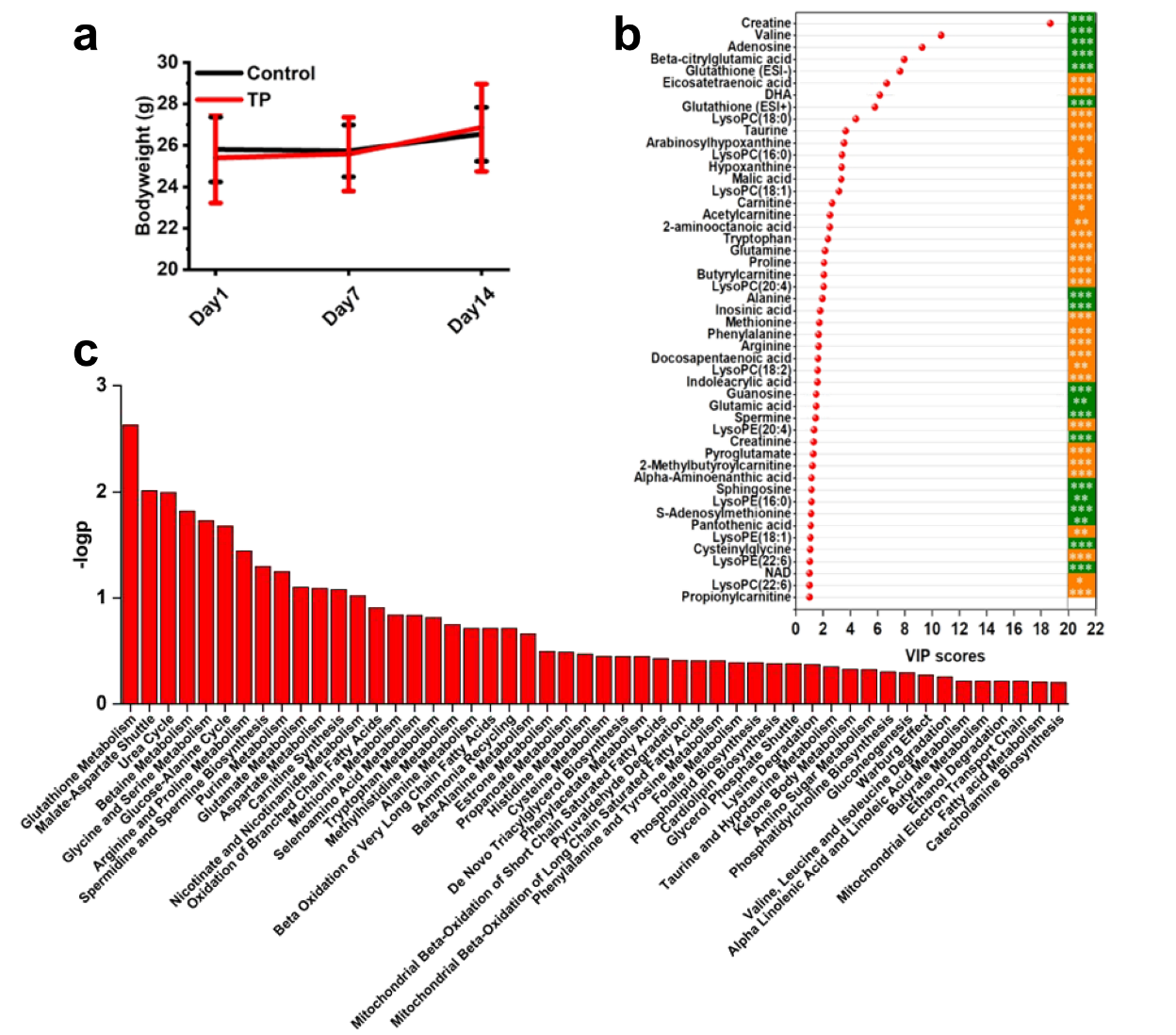


**Supplementary** **Fig. 1. Various metabolites and pathways were influenced by TP**. **a** Body weight after TP treatment. **b** VIP value and significant difference of the changed metabolites in testis. **c** Pathway enrichment of the metabolites disrupted by TP.
