# Supplementary material for: Polyamine metabolism links gut microbiota and testicular dysfunction
Source: Microbiome. 2021 Nov 11;9:224. doi: 10.1186/s40168-021-01157-z (PMC8582214; doi:10.1186/s40168-021-01157-z)
Supplement: Supplementary file 6 — Additional file 5: Supplementary Figure 2. Time-dependent effects of TP-induced testicular toxicity. Id4 for spermatogonial stem cell; C-kit for differentiated spermatogonium; Scp3 for meiotic spermatocyte; Crem for late meiotic spermatocyte and round spermatid; Acrosin for haploid cell; Vimentin for sertoli cell. [file 40168_2021_1157_MOESM6_ESM.docx]

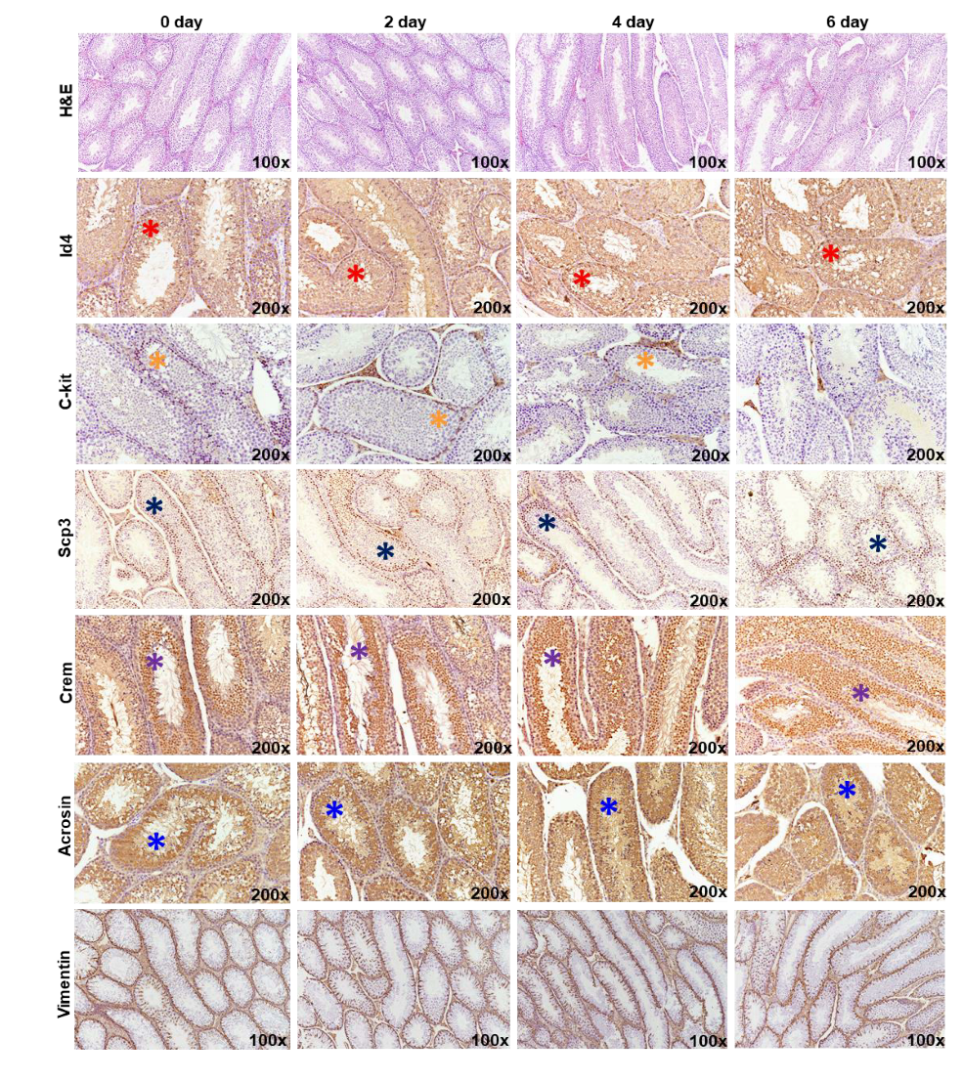

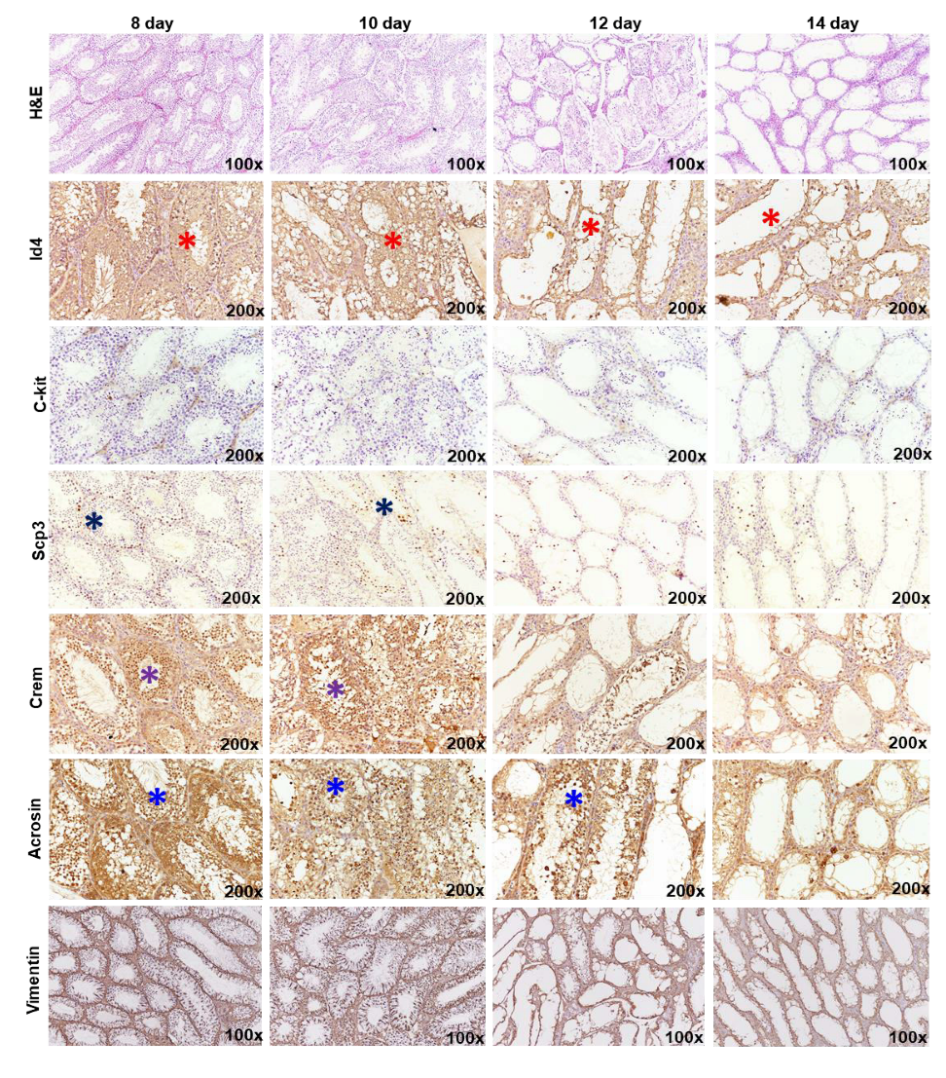


**Supplementary** **Fig. 2. Time-dependent effect of TP-induced testicular toxicity**. Id4 for spermatogonial stem cell; C-kit for differentiated spermatogonium; Scp3 for meiotic spermatocyte; Crem for late meiotic spermatocyte and round spermatid; Acrosin for haploid cell; Vimentin for sertoli cell.
