# Supplementary material for: Polyamine metabolism links gut microbiota and testicular dysfunction
Source: Microbiome. 2021 Nov 11;9:224. doi: 10.1186/s40168-021-01157-z (PMC8582214; doi:10.1186/s40168-021-01157-z)
Supplement: Supplementary file 7 — Additional file 6: Supplementary Figure 3. Polyamine pathway was influenced by TP. a mRNA levels of genes involved in spermine synthesis, metabolism and transport. b Spectrograms of spermine and spermidine. c Correlation between the testis index and levels of spermine and spermidine in testis. d Polyamine levels. *P<0.05, **P<0.01, and ***P<0.001. [file 40168_2021_1157_MOESM7_ESM.docx]

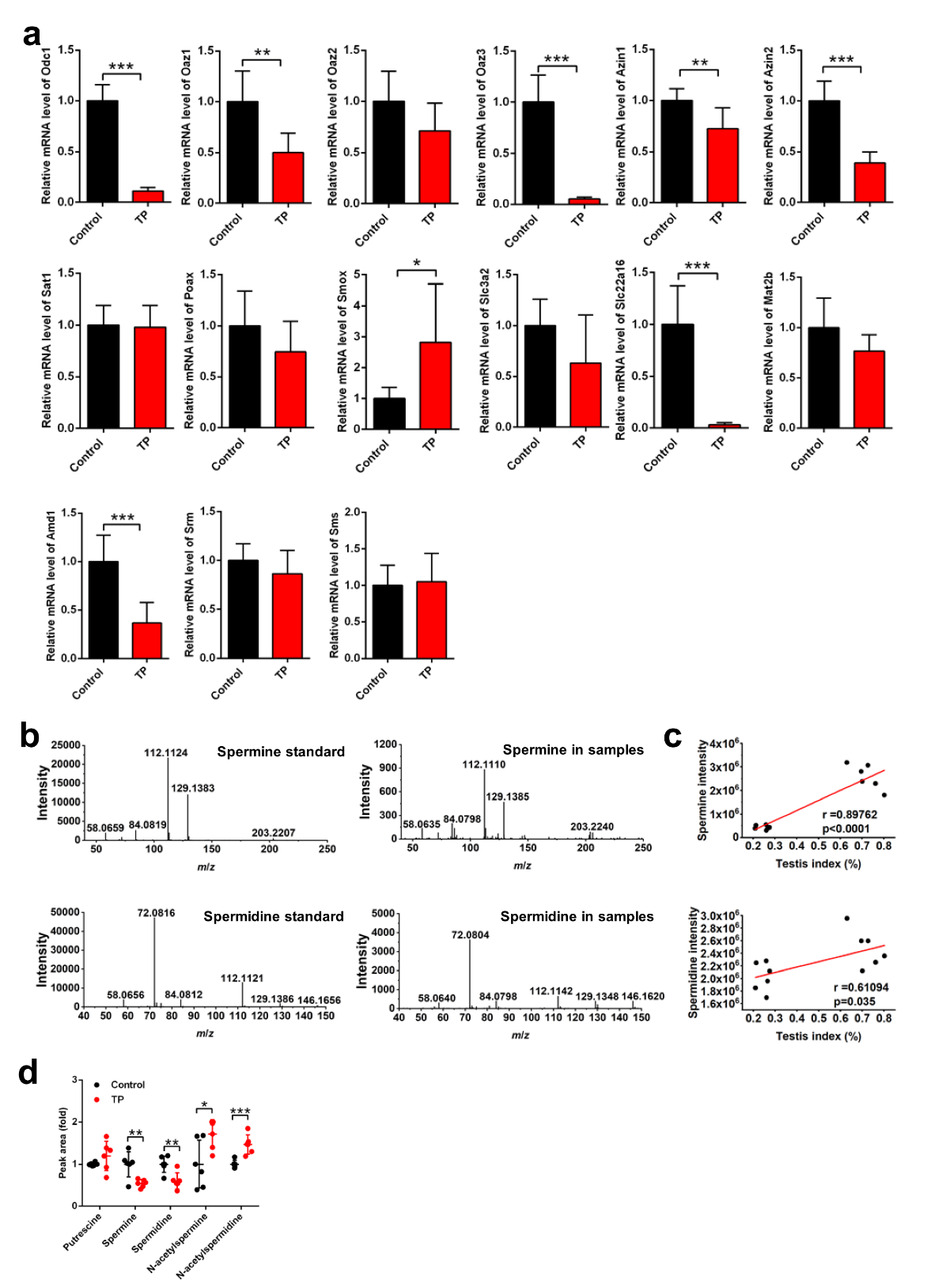


**Supplementary** **Fig. 3. Polyamine pathway was influenced by TP. a** mRNA levels of genes involved in spermine synthesis, metabolism and transport. **b** Spectrograms of spermine and spermidine. **c** Correlation between the testis index and levels of spermine and spermidine in testis. **d** Polyamine levels. **P*<0.05, ***P*<0.01, and ****P*<0.001.
