# Supplementary material for: Polyamine metabolism links gut microbiota and testicular dysfunction
Source: Microbiome. 2021 Nov 11;9:224. doi: 10.1186/s40168-021-01157-z (PMC8582214; doi:10.1186/s40168-021-01157-z)
Supplement: Supplementary file 8 — Additional file 7: Supplementary Figure 4. High-throughput sequencing results of mice treated with TP. a Relative abundance of bacterial class, order, family, genus, and species in cecum content based on 16S rRNA sequencing. b Relative abundance of parabacteroides in genus. [file 40168_2021_1157_MOESM8_ESM.docx]

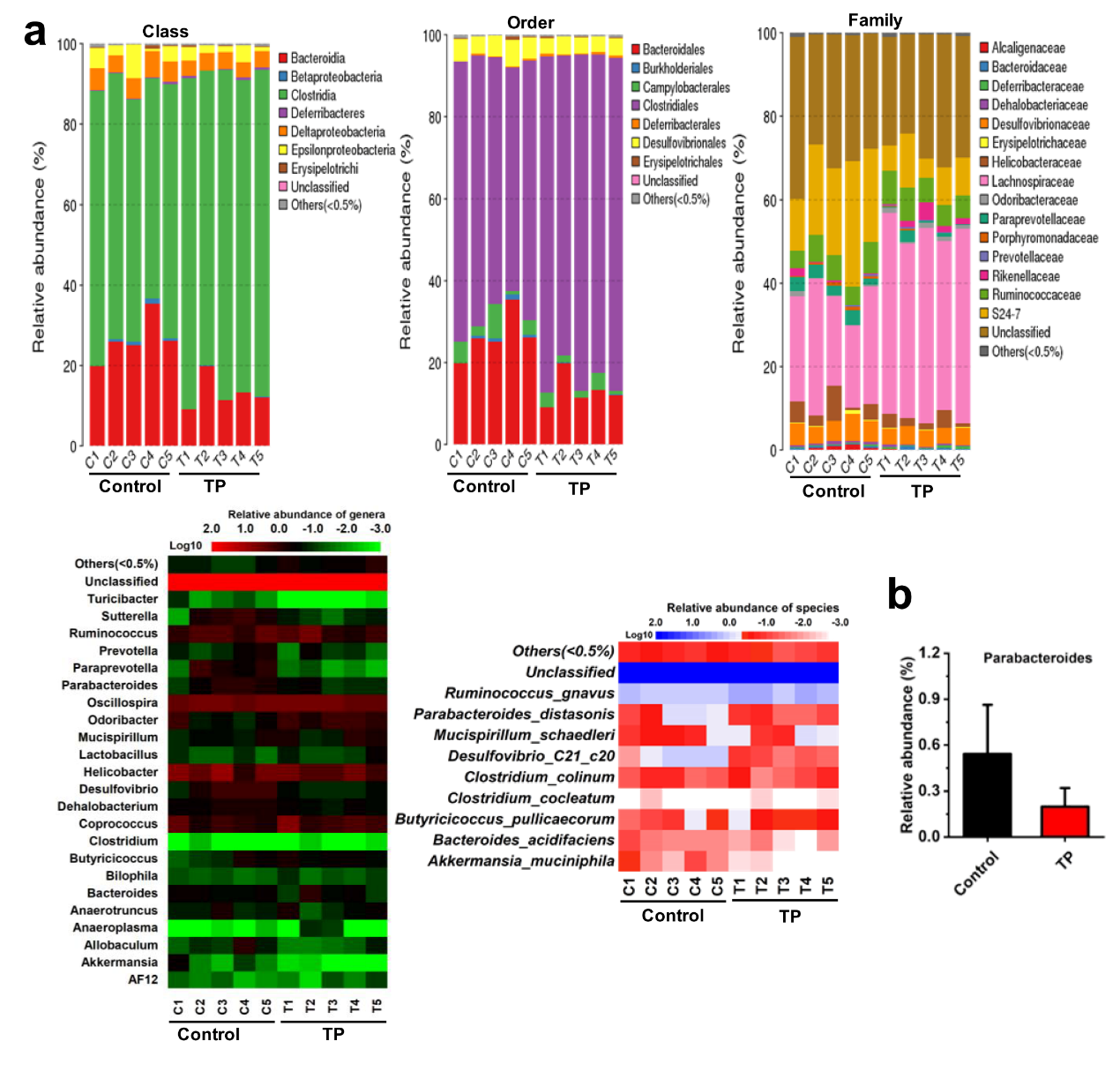


**Supplementary** **Fig. 4.** **High-throughput sequencing results of mice treated with TP. a** Relative abundance of bacterial class, order, family, genus, and species in cecum content based on 16S rRNA sequencing. **b** Relative abundance of parabacteroides in genus.
