# Supplementary material for: Polyamine metabolism links gut microbiota and testicular dysfunction
Source: Microbiome. 2021 Nov 11;9:224. doi: 10.1186/s40168-021-01157-z (PMC8582214; doi:10.1186/s40168-021-01157-z)
Supplement: Supplementary file 9 — Additional file 8: Supplementary Figure 5. The protective effect of spermine on TP-induced testicular dysfunction. a Relative intensity of polyamines in testis after supplement with exogenous spermine. b mRNA level of genes related to mitochondrial function and ATP utilization. c Body weight and tissue index (tissue weight/body weight × 100%) of the offspring. d The protective effect of spermidine on TP-induced testicular dysfunction. *P<0.05, **P<0.01, and ***P<0.001. [file 40168_2021_1157_MOESM9_ESM.docx]

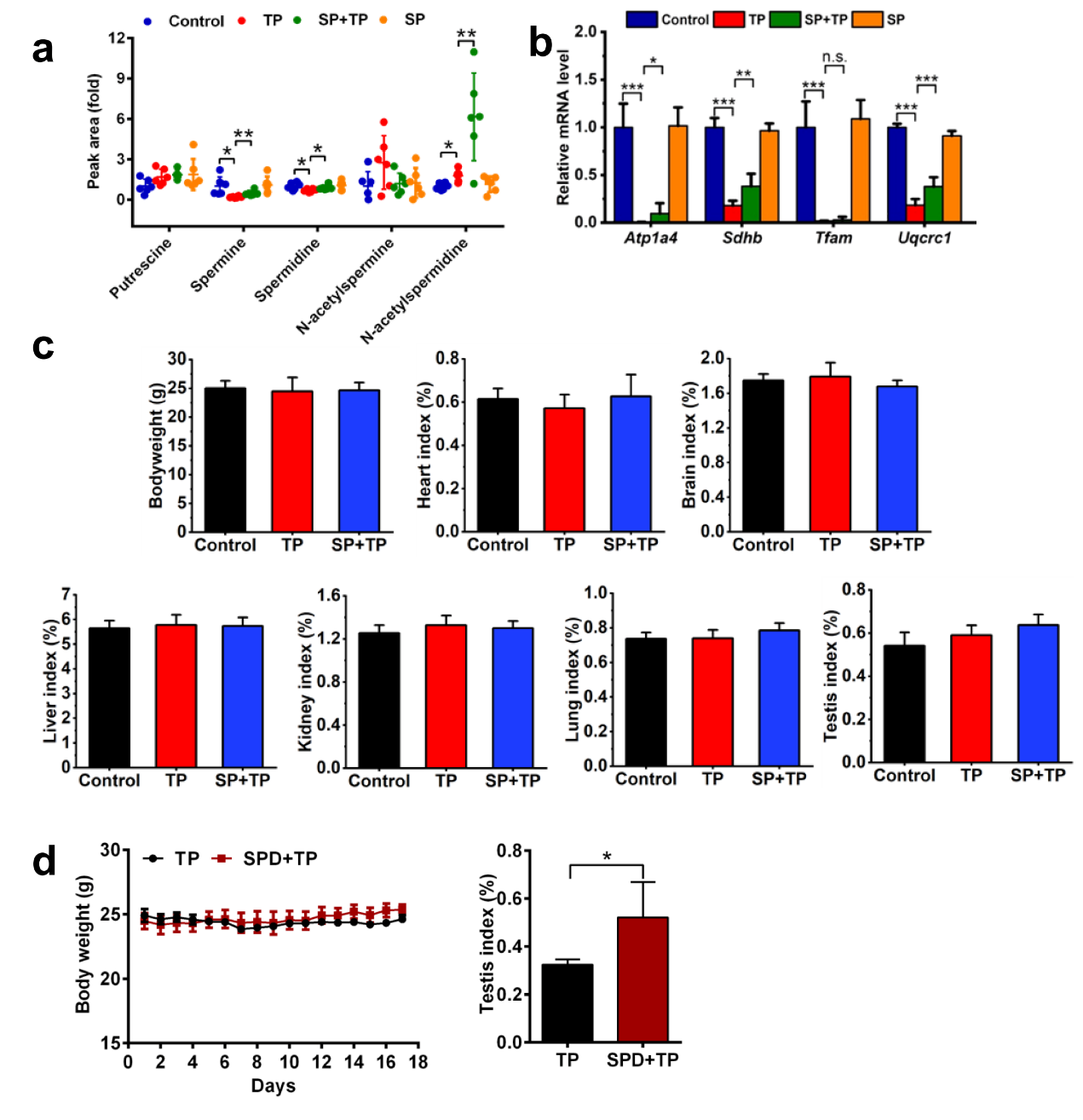


**Supplementary** **Fig. 5. The protective effect of spermine on TP-induced testicular dysfunction. a** Relative intensity of polyamines in testis after supplement with exogenous spermine. **b** mRNA level of genes related to mitochondrial function and ATP utilization. **c** Body weight and tissue index (tissue weight/body weight × 100%) of the offspring. **d** The protective effect of spermidine on TP-induced testicular dysfunction. **P*<0.05, ***P*<0.01, and ****P*<0.001.
