# Supplementary material for: Polyamine metabolism links gut microbiota and testicular dysfunction
Source: Microbiome. 2021 Nov 11;9:224. doi: 10.1186/s40168-021-01157-z (PMC8582214; doi:10.1186/s40168-021-01157-z)
Supplement: Supplementary file 10 — Additional file 9: Supplementary Figure 6. Spermine protected TP-induced testicular injury in TM4 cells. a The protective effect of spermine was evaluated by cell viability, extracellular LDH levels, and MDA concentrations in cells. b Mitochondrial injury was assessed by cellular ATP levels and expression of the mitochondrial-related genes Sdhb, Tfam, and Uqcrc1. c Representative photographs and quantitative analysis of mitochondrial membrane potential. d Eflornithine inhibited cell proliferation. e Spermine reversed the inhibited cell proliferation by eflornithine. The concentration of spermine was 6.25 μM. f Eflornithine aggravated TP-induced testicular injury. *P<0.05, **P<0.01, and ***P<0.001. [file 40168_2021_1157_MOESM10_ESM.docx]

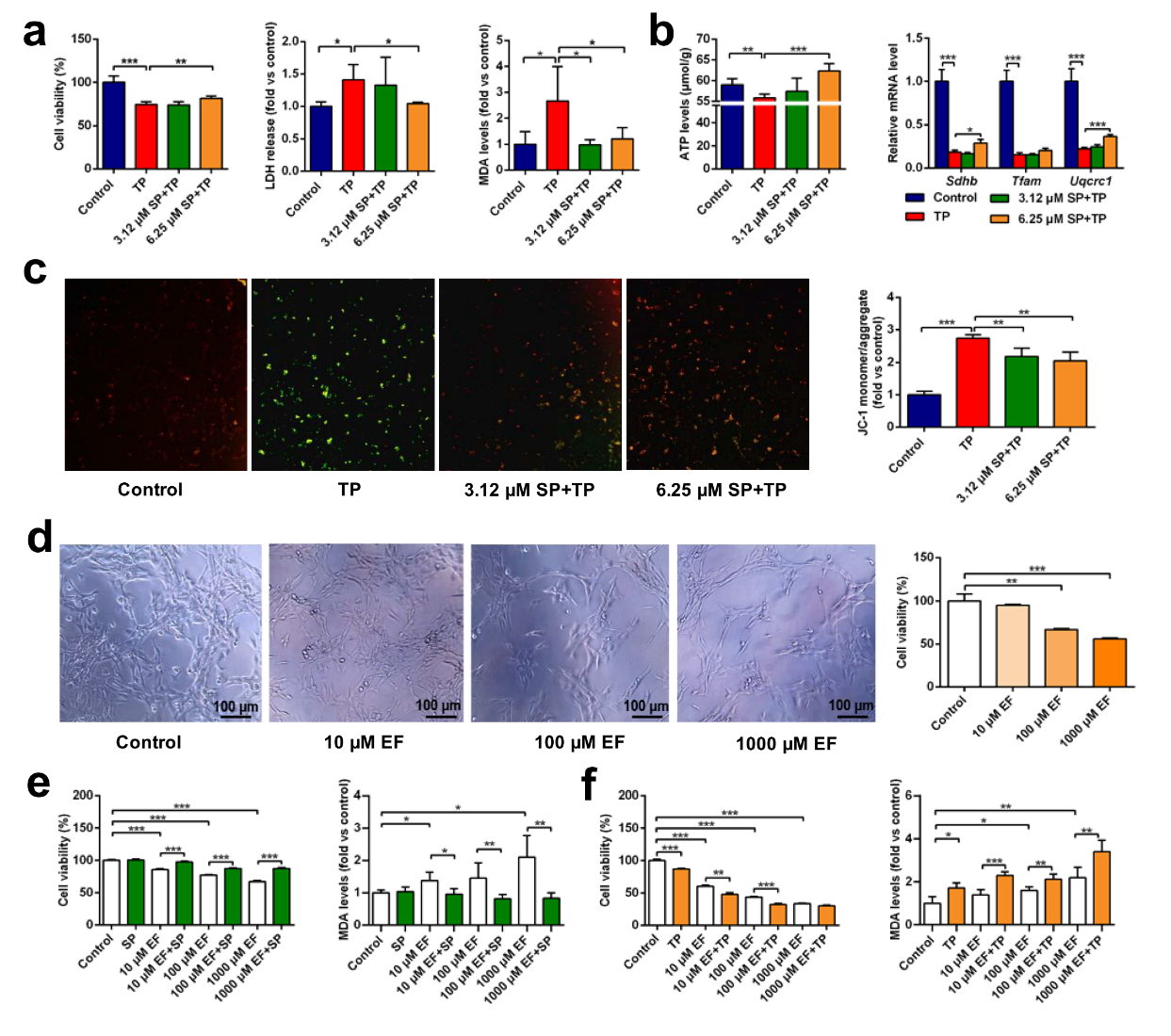


**Supplementary** **Fig. 6. Spermine protected TP-induced testicular injury in TM4 cells. a** The protective effect of spermine was evaluated by cell viability, extracellular LDH levels, and MDA concentrations in cells. **b** Mitochondrial injury was assessed by cellular ATP levels and expression of the mitochondrial-related genes *Sdhb*, *Tfam*, and *Uqcrc1*. **c** Representative photographs and quantitative analysis of mitochondrial membrane potential. **d** Eflornithine inhibited cell proliferation. **e** Spermine reversed the inhibited cell proliferation by eflornithine. The concentration of spermine was 6.25 μM. **f** Eflornithine aggravated TP-induced testicular injury. **P*<0.05, ***P*<0.01, and ****P*<0.001.
