# Supplementary material for: Polyamine metabolism links gut microbiota and testicular dysfunction
Source: Microbiome. 2021 Nov 11;9:224. doi: 10.1186/s40168-021-01157-z (PMC8582214; doi:10.1186/s40168-021-01157-z)
Supplement: Supplementary file 11 — Additional file 10: Supplementary Figure 7. Loss of gut microbiota aggravated testicular injury. a Testicular polyamine levels. b Serum LH and FSH levels. c Testosterone level and steroidogenic genes. d Spermatogonial stem cell-related genes. e Inflammatory factor and oxidative stress. *P<0.05, **P<0.01, and ***P<0.001. [file 40168_2021_1157_MOESM11_ESM.docx]

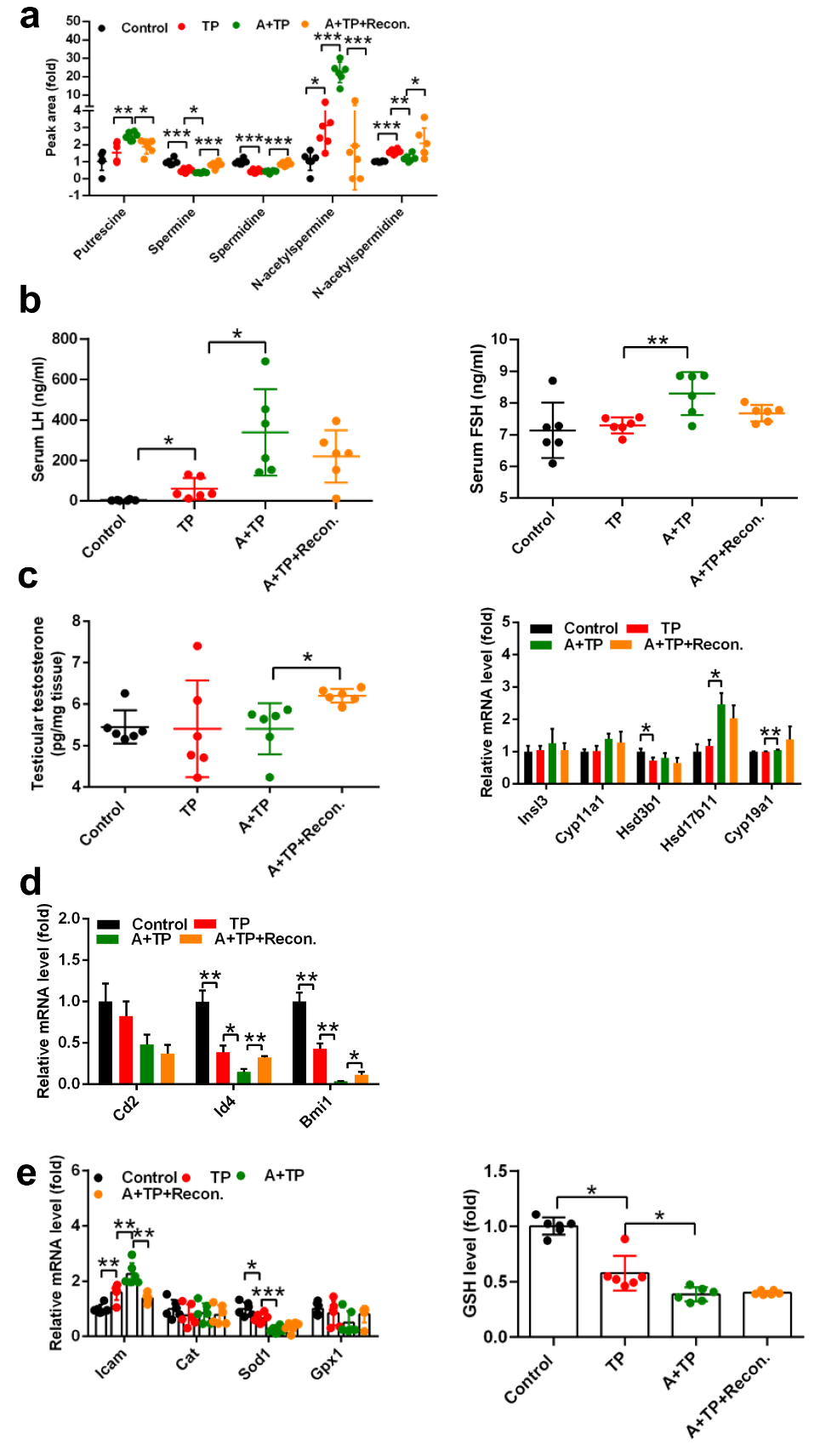


**Supplementary** **Fig. 7. Loss of gut microbiota aggravated testicular injury. a** Testicular polyamine levels. **b** Serum LH and FSH levels. **c** Testosterone level and steroidogenic genes. **d** Spermatogonial stem cell-related genes. **e** Inflammatory factor and oxidative stress. **P*<0.05, ***P*<0.01, and ****P*<0.001.
