# Supplementary material for: Polyamine metabolism links gut microbiota and testicular dysfunction
Source: Microbiome. 2021 Nov 11;9:224. doi: 10.1186/s40168-021-01157-z (PMC8582214; doi:10.1186/s40168-021-01157-z)
Supplement: Supplementary file 12 — Additional file 11: Supplementary Figure 8. mRNA level of genes related to mitochondrial function and ATP utilization. *P<0.05, **P<0.01, and ***P<0.001. [file 40168_2021_1157_MOESM12_ESM.docx]

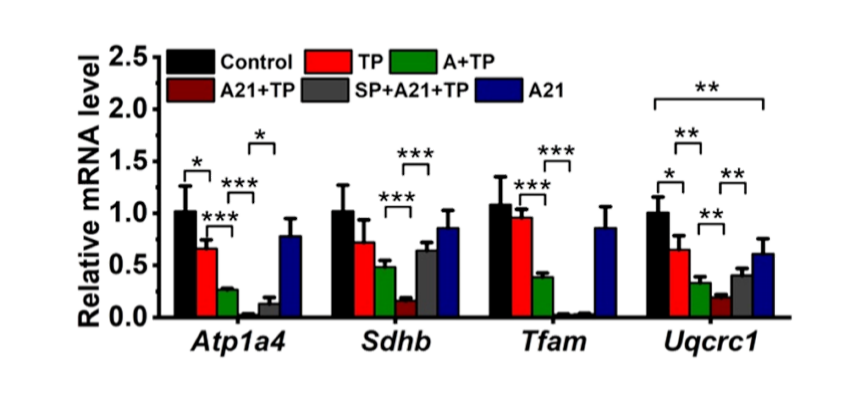


**Supplementary** **Fig. 8. mRNA level of genes related to mitochondrial function and ATP utilization.** **P*<0.05, ***P*<0.01, and ****P*<0.001.
