# Supplementary material for: Polyamine metabolism links gut microbiota and testicular dysfunction
Source: Microbiome. 2021 Nov 11;9:224. doi: 10.1186/s40168-021-01157-z (PMC8582214; doi:10.1186/s40168-021-01157-z)
Supplement: Supplementary file 13 — Additional file 12: Supplementary Figure 9. Parabacteroides distasonis protects TP-induced testicular injury. a H&E staining. b Inflammatory factor and oxidative stress. A: antibiotic; TP: triptolide; Pd.: Parabacteroides distasonis; Pd-H.: heat-killed Parabacteroides distasonis. *P<0.05, **P<0.01, and ***P<0.001. [file 40168_2021_1157_MOESM13_ESM.docx]

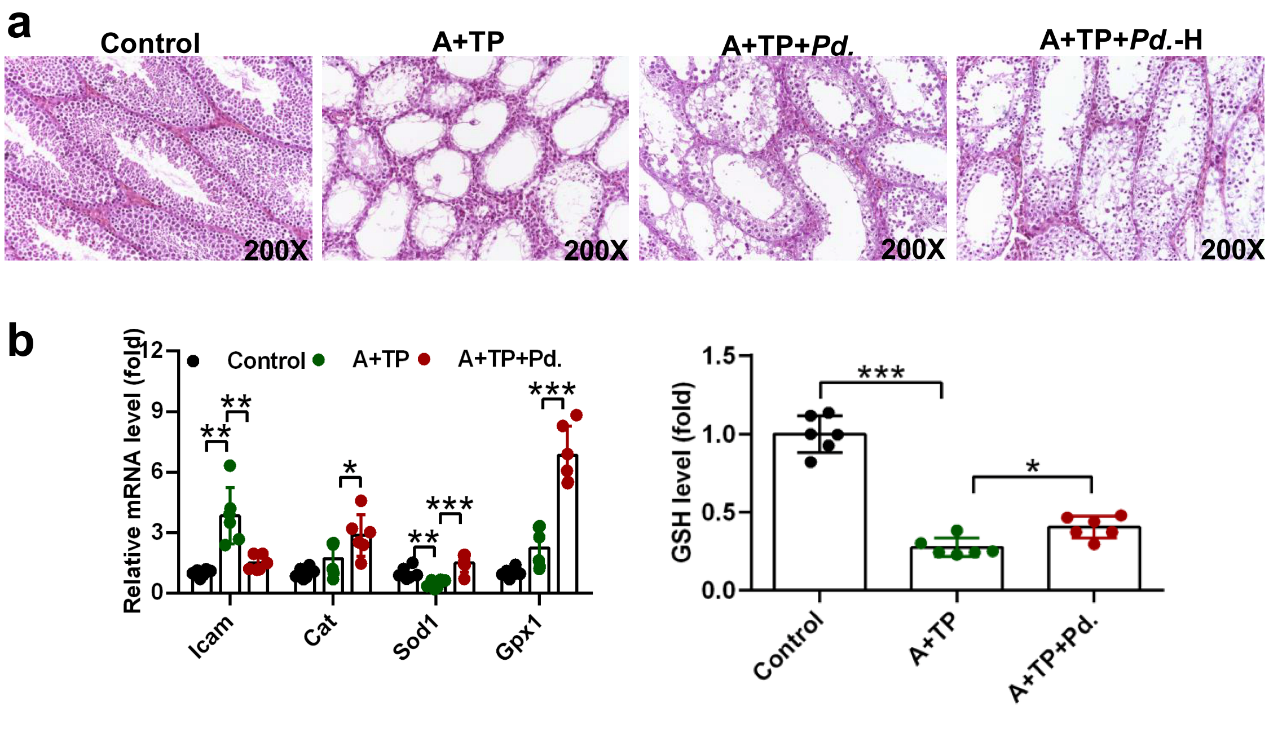


**Supplementary** **Fig. 9. *Parabacteroides distasonis* protects TP-induced testicular injury.** **a** H&E staining. **b** Inflammatory factor and oxidative stress. A: antibiotic; TP: triptolide; *Pd.*: *Parabacteroides distasonis*; *Pd*-H*.*: heat-killed *Parabacteroides distasonis*. **P*<0.05, ***P*<0.01, and ****P*<0.001.
