# Supplementary material for: Polyamine metabolism links gut microbiota and testicular dysfunction
Source: Microbiome. 2021 Nov 11;9:224. doi: 10.1186/s40168-021-01157-z (PMC8582214; doi:10.1186/s40168-021-01157-z)
Supplement: Supplementary file 14 — Additional file 13: Supplementary Methods (DOCX 18 kb) [file 40168_2021_1157_MOESM14_ESM.docx]

**Supplementary Methods**

**ODC activity**

ODC activity was evaluated based on previous study [1]. The experiment was assigned into 8 groups (n=6): 0, 40, 160, 640 nM TP groups (background correction); 0, 40, 160, 640 nM TP + ornithine groups. 100 mg mice testis were minced in 1000 µL buffer (20 mM sodium phosphate, 0.2 mM EDTA, and 0.1 mM 5''-pyridoxal phosphate, PH 7.4) at 4℃. After centrifugation at 12000 rpm for 20 min at 4℃, supernatant was transferred to a new Eppendorf vial. The protein level was measured using protein quantification Kit (about 6 mg/mL protein, Beyotime, China).

The 150 µL assay mixture containing 100 µL supernatant (about 600 µg protein), 50 µL water, and 0, 40, 160, 640 nM TP was incubated at 37℃ for 30 min (300 rpm). Ornithine (50 µL, 50 µM) was added and the reaction was allowed to proceed for 60 min. The absence of ornithine in the incubation system was used to background correction. The reaction was stopped by adding 400 µL of ice-cold acetonitrile. The solution was centrifuged at 15000 rpm for 10 min following vortexing for 1 min. A 5 µL aliquot of the supernatant was injected into a UPLC-ESI-QTOFMS system for analysis. Putrescine and ornithine levels were obtained based on target analysis and standard curve. ODC activity = [(putrescine - putrescine background) / (ornithine – ornithine background)] × 100 (%).

**SSAT activity**

SSAT activity was evaluated based on previous study [2]. The experiment was assigned into 8 groups (n=6): 0, 40, 160, 640 nM TP groups (background correction); 0, 40, 160, 640 nM TP + spermidine groups. 100 mg testis were minced in 200 µL buffer (0.1 M Tris-HCl, 100 µM acetyl-CoA, PH 7.8). After centrifugation at 12000 rpm for 20 min at 4℃, supernatant was transferred to a new Eppendorf vial. The protein content was measured using protein quantification Kit (about 3 mg/mL, Beyotime, China).

The 200 µL assay mixture containing 200 µL supernatant and TP (0, 40, 160, 640 nM) was incubated at 37℃ for 30 min (300 rpm). Spermidine (2 µL, 40 µM) was added and the reaction was allowed to proceed for 60 min. The absence of spermidine in the incubation system was used to background correction. The reaction was stopped by adding 600 µL of ice-cold acetonitrile. The solution was centrifuged at 15000 rpm for 10 min following vortexing for 1 min. A 5 µL aliquot of the supernatant was injected into a UPLC-ESI-QTOFMS system for analysis. Spermidine and N-acetylspermidine levels were obtained based on target analysis and standard curve. SSAT activity = [(N-acetylspermidine – N-acetylspermidine background) / (spermidine – spermidine background)] × 100 (%).

**UPLC-ESI-QTOF analysis conditions for ODC and SSAT activities**

Putrescine, ornithine, N-acetylspermidine, and spermidine were separated using a UPLC system containing a 1290 Autosampler and 1290 Quat Pump (Aglient, Santa Clara, CA) equipped with XDB-C18 column (2.1×100 mm, 1.8 µM). Column temperature was maintained at 45°C. The flow rate was 0.3 mL/min with a gradient ranging from 2% to 98% acetonitrile containing 0.1% formic acid in 16 min run.

The mass signals of ion were collected in positive mode by the electrospray ionization 6530 QTOFMS (Agilent, Santa Clara, CA). Nitrogen was applied as both drying gas (9 L/min) and the collision gas. The drying gas temperature was set at 350°C and nebulizer pressure was kept at 35 psi. Capillary voltage was set at 3.5 kV. Chromatographic and spectral data of samples were acquired by MassHunter Workstation data Acquisition software (Aglient, Santa Clara, CA).

**References**

1. Badolo L, Berlaimont V, Helson-Cambier M, Hanocq M, Dubois J. Simple and rapid enzymatic assay of ornithine decarboxylase activity. *Talanta* 1999,**48**:127-134.

2. Takao K, Miyatake S, Fukazawa K, Wada M, Sugita Y, Shirahata A. Measurement of spermidine/spermine-N1-acetyltransferase activity by high-performance liquid chromatography with N1-dansylnorspermine as the substrate. *Anal Biochem* 2008,**376**:277-279.
